# The Contextual Resonance Theory of Psychopathology: A Cognitive–Systemic Framework for Transgenerational Trauma, Distributed Pathology, and Corrective Recontextualization

**DOI:** 10.3390/bs16081404

**Published:** 2026-08-16

**Authors:** Narcisa Carmen Mladin, Dana Rad, Loredana Ileana Vîșcu, Ioana Eva Cădariu, Radiana Marcu, Daniela Roman, Gavril Rad

**Affiliations:** 1 Department of Internal Medicine, “Vasile Goldis” Western University of Arad, 310025 Arad, Romania; mladin_narcisa@yahoo.com; 2 Center of Research Development and Innovation in Psychology, Faculty of Educational Sciences Psychology and Social Work, Aurel Vlaicu University of Arad, 310025 Arad, Romania; radi.marcu@yahoo.com (R.M.); dana.rad@uav.ro (G.R.); 3 Department of Psychology, Tibiscus University of Timisoara, 300223 Timisoara, Romania; loredana.viscu@gmail.com (L.I.V.); cadariuioanaeva@yahoo.com (I.E.C.); 4 Institute of Psychotherapy Psychological Counselling and Clinical Supervision, 300223 Timisoara, Romania; 5 Department of Psychology, Faculty of Socio-Humanistic Sciences, University of Oradea, 410087 Oradea, Romania

**Keywords:** transgenerational trauma, psychopathology, predictive processing, contextual resonance, distributed pathology, cognitive systems, trauma transmission, ecological psychology, mental health, corrective recontextualization

## Abstract

The transmission of trauma across generations has traditionally been explained through genetic, epigenetic, familial, and attachment-based mechanisms. While these approaches have generated valuable insights, they remain insufficient for explaining the persistence of psychological vulnerability in contexts where the original traumatic event has disappeared but maladaptive patterns continue to emerge across generations. This paper introduces the Contextual Resonance Theory of Psychopathology (CRTP), a novel cognitive–systemic framework proposing that transgenerational trauma is maintained through the recursive reproduction of trauma-shaped environments rather than exclusively through biological inheritance. According to this model, traumatic experiences generate adaptive cognitive, emotional, and behavioral responses that become embedded within family narratives, relational structures, institutional practices, and cultural expectations. Over time, these adaptations evolve into self-maintaining contextual systems that continuously communicate threat, unpredictability, and hypervigilance to subsequent generations. The theory introduces the concepts of contextual traumatic inertia, distributed psychopathology, and recursive contextual resonance to explain how vulnerability becomes socially reproduced even in the absence of direct trauma exposure. Drawing on predictive processing, ecological psychology, systems theory, and contemporary trauma research, the paper proposes that psychopathology emerges from persistent interactions between individuals and pathogenic environments rather than solely from intrapsychic dysfunction. Furthermore, a complementary mechanism termed corrective recontextualization is proposed as a pathway for reversing transgenerational vulnerability through repeated exposure to safe, predictable, and psychologically restorative contexts. The framework offers an integrative perspective capable of bridging cognitive neuroscience, developmental psychopathology, trauma studies, and mental health intervention research. Clinical, educational, organizational, and societal implications are discussed, alongside future directions for empirical validation and neurocognitive investigation.

## 1. Introduction

### 1.1. The Challenge of Explaining Transgenerational Trauma

Traumatic experiences have traditionally been conceptualized as events that affect the psychological functioning of directly exposed individuals. However, over the past several decades, evidence emerging from studies of war survivors, genocide victims, refugees, Indigenous communities, and families affected by chronic violence has challenged this assumption. Increasingly, researchers have observed that the psychological consequences of traumatic experiences often appear in descendants who were never directly exposed to the original traumatic event. This phenomenon, commonly referred to as transgenerational or intergenerational trauma, has become one of the most complex and debated topics in contemporary trauma research.

Early observations emerged primarily from studies involving Holocaust survivors and their offspring. Researchers noticed elevated levels of anxiety, hypervigilance, emotional dysregulation, and identity-related difficulties among descendants despite the absence of direct trauma exposure (Kellermann, 2001). Similar patterns were later documented among survivors of war, colonization, political oppression, forced displacement, and mass violence (Atkinson et al., 2010; Harkness, 1993; Maxwell, 2014). These findings raised a fundamental question that continues to challenge psychology, psychiatry, and neuroscience: How can an event experienced by one generation continue to influence the mental health of subsequent generations?

The difficulty of answering this question stems partly from the fact that trauma does not operate exclusively at a single level of human functioning. It affects biological systems, cognitive schemas, emotional regulation processes, attachment relationships, family interactions, collective narratives, and sociocultural structures simultaneously. Consequently, no single explanatory framework has been able to fully account for the remarkable persistence of trauma-related vulnerability across generations.

Krippner and Barrett (2019) argue that the field has often struggled with the tendency to conceptualize trauma transmission as an almost automatic process, insufficiently considering the historical and social conditions through which traumatic meanings are continuously reconstructed. Similarly, Froń et al. (2026) note that transgenerational trauma remains a multidimensional phenomenon involving overlapping psychological, biological, interpersonal, and cultural mechanisms. Contemporary reviews continue to emphasize that although substantial empirical evidence supports the existence of transgenerational effects, consensus regarding the underlying mechanisms remains elusive (Lehrner & Yehuda, 2018).

At the same time, the phenomenon cannot be reduced solely to pathology. Studies conducted among descendants of Holocaust survivors demonstrate that alongside vulnerability, resilience, meaning-making, adaptive identity formation, and posttraumatic growth may also be transmitted across generations (Maxwell, 2014; R. D. Goodman, 2013). Through the development of the transgenerational trauma and resilience genogram, research further illustrates that family histories frequently contain both traumatic legacies and resources for adaptation, suggesting that transmission processes are considerably more complex than simple models of vulnerability inheritance would imply (Danieli et al., 2016).

This complexity has generated a rich but fragmented body of theories attempting to explain how trauma crosses generational boundaries.

While theoretical diversity has substantially advanced knowledge regarding transgenerational trauma, the coexistence of multiple explanatory frameworks also generates an important conceptual challenge. Contemporary theories typically concentrate on particular mechanisms—such as biological inheritance, attachment processes, family interactions, ecological influences, or predictive cognition, without providing an overarching explanation of how these mechanisms interact across levels of analysis. Importantly, this does not imply that existing theories are incomplete or inadequate. On the contrary, each offers valuable insight into specific dimensions of trauma transmission. However, many clinically and theoretically relevant questions emerge precisely at the interfaces between these explanatory traditions. For example, how do biological vulnerability, relational experiences, institutional practices, and sociocultural environments dynamically reinforce one another across generations? How do adaptive processes operating at one systemic level become stabilized and reproduced at another? Addressing such questions requires not the replacement of existing theories but an integrative framework capable of organizing their complementary contributions within a coherent explanatory architecture. The motivation for the present theory therefore lies not in the existence of multiple theories per se, but in the absence of a conceptual model explicitly describing how their principal mechanisms interact within a recursive multilevel system.

### 1.2. Literature Approach and Conceptual Scope

The present manuscript is conceived as a theoretical and integrative conceptual paper, whose primary objective is to develop the Contextual Resonance Theory of Psychopathology (CRTP). Accordingly, the literature review is not intended to constitute a systematic or exhaustive synthesis of all available evidence on transgenerational trauma. Rather, its purpose is to identify, examine, and conceptually integrate the principal theoretical traditions that have shaped current understanding of trauma transmission across generations.

The literature included in this review was selected purposively to represent the major explanatory perspectives relevant to the proposed framework. These perspectives comprise historical and cultural approaches, genetic and epigenetic models, attachment theory, family systems approaches, ecological and socioecological frameworks, resilience research, and predictive processing. Within each tradition, influential theoretical contributions were considered alongside contemporary empirical studies that illustrate recent developments, unresolved questions, and areas of convergence relevant to the present conceptual synthesis. Particular attention was also given to recent theoretical developments concerning intergenerational trauma, including integrative therapeutic frameworks, systematic reviews and meta-analyses, philosophical approaches to transgenerational embodiment, trauma-responsive peacebuilding, and contemporary analyses of family memory and cultural transmission (Bowe et al., 2025; Butt, 2025; de Ávila, 2025; Reimann & Clarke-Habibi, 2026; Zasiekina et al., 2026).

Consequently, the review should be interpreted as illustrative and theory-building rather than systematic. Its function is to establish the conceptual foundations necessary for developing the proposed framework, to identify the explanatory limitations of existing models, and to justify the need for an integrative cognitive–systemic perspective. The objective is therefore not to evaluate the entire body of evidence concerning transgenerational trauma, but to synthesize representative theoretical contributions that collectively support the formulation of the Contextual Resonance Theory of Psychopathology. Accordingly, both foundational theoretical contributions and recent conceptual advances were incorporated to ensure that the proposed framework reflects the current state of knowledge while addressing unresolved theoretical questions in the field (Bowe et al., 2025; Butt, 2025; de Ávila, 2025; Reimann & Clarke-Habibi, 2026; Zasiekina et al., 2026).

### 1.3. Current Models of Trauma Transmission

Some of the earliest attempts to explain transgenerational trauma emerged from historical, cultural, and community-based perspectives, which challenged the notion that trauma is confined to the psychological functioning of directly exposed individuals. Rather than conceptualizing trauma as an isolated intrapsychic wound, these approaches view it as a collective disruption capable of reshaping cultural memory, social identity, family life, and community functioning across generations.

Recent scholarship has further demonstrated that transgenerational trauma is preserved not only through historical narratives but also through family memory, identity construction, and cultural storytelling. Examining diasporic women’s literature from the Global South, Butt (2025) illustrates how family memories become intertwined with collective histories, allowing trauma to persist across generations even in the absence of direct exposure. These observations reinforce the view that trauma transmission is inseparable from the cultural contexts in which personal and collective identities are continuously reconstructed.

This perspective is particularly evident in the work of Atkinson et al. (2010), who examined the experiences of Aboriginal and Torres Strait Islander communities. Their analysis demonstrated that the consequences of colonization, forced displacement, and systemic marginalization extend far beyond the individuals who initially endured these experiences. Trauma becomes embedded within collective narratives, community structures, and everyday social realities, contributing to enduring patterns of vulnerability that continue to influence wellbeing long after the original traumatic events have passed. In this sense, trauma is not merely remembered; it becomes woven into the social fabric of communities.

A similar argument was advanced by Harkness (1993), who emphasized that war-related trauma frequently survives through changes in family relationships, emotional communication, and culturally transmitted perceptions of threat. The effects of violence and loss are often carried forward through everyday interactions, parenting practices, and shared assumptions about safety and danger. Importantly, this transmission does not require explicit discussions of past traumatic events; it may occur through subtle relational and cultural processes that shape how subsequent generations interpret and respond to the world around them.

Research conducted among Brazilian descendants of Holocaust survivors further illustrates the complexity of these processes (Braga et al., 2012). Their findings revealed that family narratives often contain both trauma and resilience, suggesting that what is transmitted across generations is not solely suffering but also adaptive capacities, survival strategies, and sources of meaning. This observation challenges deficit-oriented perspectives by highlighting that traumatic legacies may simultaneously convey vulnerability and strength.

Building on these earlier observations, researchers proposed one of the most comprehensive frameworks of multigenerational trauma, arguing that transmission occurs through multiple interconnected pathways (Danieli et al., 2016). Biological, psychological, familial, social, and cultural mechanisms do not operate independently but interact dynamically across generations, creating complex patterns of risk and adaptation. More recently, Starrs and Békés (2025) extended this line of thinking by developing a historical intergenerational trauma transmission model that integrates parental functioning, family processes, offspring vulnerability, and broader sociocultural influences into a unified explanatory framework.

The importance of contextual influences was further reinforced by Kahn and Denov (2022) in their study of Rwandan genocidal rape survivors and their children. Drawing on a culturally enhanced bioecological perspective, they demonstrated that trauma transmission cannot be adequately understood without considering the continuous interaction between individual experiences, family relationships, community environments, and culturally embedded systems of meaning. Their findings suggest that trauma unfolds within an ecological network of influences rather than through isolated psychological or biological mechanisms.

Reflecting broader developments within the field, Salberg (2024) notes that contemporary theories increasingly recognize trauma as a multilevel phenomenon that cannot be reduced to intrapsychic processes alone. The evolution of trauma scholarship has progressively shifted attention from individual pathology toward more complex models that acknowledge the interplay between personal experiences, relational dynamics, historical forces, and sociocultural environments. Together, these perspectives provide a foundation for understanding trauma as a phenomenon that is not only carried by individuals but also sustained, transformed, and reproduced within the contexts in which individuals and communities live.

Sociological perspectives further enrich the understanding of transgenerational trauma by explaining how traumatic adaptations become embedded within enduring social structures. Particularly relevant in this regard is Bourdieu’s theory of practice, according to which social life is organized through the dynamic interaction of field, habitus, and different forms of capital (Bourdieu, 1977, 1990). Within this perspective, traumatic experiences may gradually contribute to the formation of a trauma-shaped habitus, namely a system of durable dispositions through which individuals perceive danger, interpret uncertainty, regulate emotions, and engage in social relationships. These dispositions are neither exclusively psychological nor biologically determined but develop through repeated participation in social fields characterized by violence, exclusion, instability, or chronic insecurity. Recent scholarship has extended this perspective by proposing that trauma itself may function as a form of cultural capital, influencing identity construction, social recognition, and access to symbolic resources while simultaneously shaping collective narratives about suffering, resilience, and belonging (S. Goodman, 2024). Critical feminist scholarship further emphasizes that trauma is often reproduced through unequal power relations, gender hierarchies, institutional exclusion, and structural marginalization, demonstrating that vulnerability is maintained not only through interpersonal interactions but also through historically situated systems of domination (S. Goodman, 2024). These sociological perspectives complement psychological and biological models by illustrating how social structures participate in the persistence of trauma across generations. Within the Contextual Resonance Theory of Psychopathology, however, such processes are conceptualized as components of broader trauma-shaped contexts that interact recursively with biological, cognitive, relational, institutional, and cultural mechanisms, thereby contributing to the long-term continuity or transformation of psychopathological vulnerability.

#### 1.3.1. Genetic Perspectives

Genetic explanations of transgenerational trauma emerged from efforts to determine whether vulnerability to trauma-related psychopathology could be partially inherited through biological predispositions. Rather than suggesting that traumatic experiences themselves are genetically transmitted, these perspectives propose that certain inherited characteristics may increase susceptibility to developing psychological difficulties following exposure to adversity. Research within behavioral genetics has therefore focused on identifying heritable factors associated with emotional reactivity, stress sensitivity, impulse control, and affect regulation.

Within this framework, Hines and Saudino (2002) examined the intergenerational transmission of intimate partner violence and argued that inherited temperamental traits may contribute to the recurrence of maladaptive relational patterns across generations. Complementing this behavioral genetic perspective, Yehuda and Lehrner (2018) highlighted the potential role of epigenetic mechanisms in the intergenerational transmission of trauma effects, suggesting that biological adaptations associated with traumatic exposure may represent an additional pathway through which trauma-related vulnerability can extend across generations. Their work suggested that biological predispositions could influence how individuals perceive, regulate, and respond to emotionally charged situations, thereby increasing the likelihood of reproducing dysfunctional interpersonal behaviors observed within the family environment. From this perspective, genetic factors do not directly transmit trauma but may shape an individual’s vulnerability to its psychological consequences.

Genetic perspectives provide important insights into biological vulnerability but cannot, on their own, explain the persistence and variability of transgenerational trauma. Contemporary research increasingly recognizes that genetic predispositions interact dynamically with developmental, relational, and contextual influences.

#### 1.3.2. Epigenetic Inheritance

The emergence of epigenetics has significantly reshaped contemporary understandings of transgenerational trauma by offering a potential mechanism through which environmental experiences may influence biological functioning across generations without altering the underlying DNA sequence. Unlike traditional genetic models, which focus on inherited genetic variants, epigenetic approaches emphasize the dynamic ways in which environmental exposures, including traumatic experiences, may modify patterns of gene expression, potentially affecting how individuals respond to stress, regulate emotions, and adapt to adversity.

Among the most influential contributors to this line of research, Jawaid et al. (2018) proposed that exposure to severe trauma may induce biological alterations within stress-regulation systems, particularly those involving glucocorticoid signaling and the hypothalamic–pituitary–adrenal (HPA) axis. Their studies involving Holocaust survivors and their offspring generated considerable interest by suggesting that some trauma-related biological adaptations may be detectable not only in directly exposed individuals but also in subsequent generations. These findings contributed to a growing body of literature exploring how traumatic experiences may become biologically embedded and potentially influence developmental trajectories beyond the original exposure.

Expanding this perspective, R. D. Goodman (2013) argued that biological mechanisms should not be examined independently of the sociocultural environments in which trauma occurs. They proposed that cultural trauma and biological adaptation may operate in parallel, creating complex pathways through which collective historical experiences influence individual development. In this view, trauma transmission reflects an ongoing interaction between biological processes and the social worlds that give meaning to traumatic events.

Similarly, Ghai and Kader (2022) described transgenerational epigenetics as a promising conceptual bridge between environmental experiences and biological inheritance. Their work highlights the possibility that traumatic stress may leave molecular traces capable of influencing subsequent generations, while simultaneously acknowledging that many of the underlying mechanisms remain insufficiently understood. Rather than presenting epigenetic inheritance as an established fact, they emphasize the need for continued investigation into the biological, developmental, and environmental conditions under which such processes may occur.

Despite the growing enthusiasm surrounding epigenetic explanations, subsequent reviews have adopted a more cautious position. Ghai and Kader (2022) concluded that although the available evidence supports the plausibility of experience-dependent epigenetic effects, demonstrating true transgenerational inheritance in humans remains methodologically challenging. The complexity of separating biological influences from shared environmental and social experiences continues to represent a major obstacle for the field. Likewise, Chou et al. (2024) emphasized that PTSD-related epigenetic pathways involve intricate interactions among genetic susceptibility, environmental exposures, developmental timing, and ongoing life experiences, making simple causal explanations inadequate.

From a social work and applied perspective, Gill et al. (2026) further argued that epigenetic findings should be interpreted within broader developmental, relational, and sociocultural contexts. They caution against reducing trauma transmission to molecular mechanisms alone, emphasizing that biological processes unfold within environments that continuously shape and modify their expression throughout the lifespan. This need for interpretive caution is further reinforced by Yehuda et al. (2018), who examined the public reception of proposed epigenetic mechanisms in the transgenerational effects of trauma and highlighted the importance of distinguishing emerging scientific evidence from broader and potentially overstated interpretations of epigenetic inheritance.

While the possibility that traumatic experiences may influence biological functioning across generations is scientifically compelling, Yehuda et al. (2018) argue that public discourse often exaggerates both the certainty of existing evidence and the permanence of these effects. Epigenetic modifications are not necessarily fixed or irreversible, nor do they imply that descendants are biologically destined to reproduce the psychological consequences of their ancestors’ trauma. Instead, current evidence increasingly suggests that biological, psychological, relational, and environmental factors interact dynamically, reinforcing the need for integrative models capable of capturing the complexity of trauma transmission across generations.

#### 1.3.3. Attachment-Based Explanations

Among the psychological frameworks proposed to explain the transmission of trauma across generations, attachment theory has become one of the most influential and empirically supported perspectives. At its core, attachment theory suggests that traumatic experiences shape the quality of caregiving relationships, the caregiver’s capacity for emotional attunement, and the development of internal working models through which individuals come to understand themselves, others, and the world. Because these relational templates are established early in life and tend to guide future interpersonal functioning, trauma may be transmitted not only through explicit narratives or behaviors but also through the relational environments in which children develop.

A central contribution to this perspective was offered by Liotti (2006), who argued that unresolved attachment trauma may create a profound relational paradox in which caregivers simultaneously represent both safety and threat. For children, this contradiction generates competing motivational tendencies, seeking proximity to the caregiver while also fearing the very source of protection. Such disorganized relational experiences can contribute to dissociative processes, fragmented self-representations, and enduring difficulties in emotional regulation, thereby creating conditions through which the effects of trauma may extend across generations (Liotti, 2006).

Building on these ideas, Salberg (2016) introduced the concept of traumatic attachment, emphasizing that trauma is often transmitted through relational experiences characterized by simultaneous presence and absence. In these families, caregivers may be physically available yet emotionally inaccessible, burdened by unresolved grief, fear, or psychological pain. Trauma resides not only in what is openly communicated but also in what remains unspoken, unprocessed, and psychologically unavailable (Salberg, 2016). The emotional silences surrounding traumatic experiences may become as influential as the events themselves, shaping the developmental environment of subsequent generations.

Empirical research has provided further support for the role of attachment processes in trauma transmission. Meulewaeter et al. (2019), examining mothers with histories of trauma and substance use disorders, demonstrated how unresolved traumatic experiences may compromise caregiving capacities, emotional responsiveness, and relational stability. These disruptions create developmental contexts in which children are more likely to experience insecurity, emotional dysregulation, and difficulties in forming stable attachment relationships. Similarly, Kostova and Matanova (2024) identify attachment insecurity as one of the primary pathways through which parental trauma histories influence offspring vulnerability, highlighting the importance of relational patterns in mediating the long-term effects of traumatic experiences.

More recently, Farina and Schimmenti (2025) have argued that attachment trauma should be considered a distinct psychopathological domain rather than merely a subtype of relational adversity. This relational understanding is also consistent with family systems perspectives, which emphasize that intergenerational trauma is embedded within patterns of family organization, communication, and relational functioning that may persist across generations (Abrams, 1999). Their conceptualization positions attachment trauma at the center of multiple developmental processes, influencing self-organization, affect regulation, identity formation, interpersonal functioning, and vulnerability to a broad range of psychological difficulties. From this perspective, the enduring impact of trauma cannot be fully understood without examining how traumatic experiences become embedded within attachment relationships and subsequently shape the developmental architecture of the self.

Attachment-based explanations suggest that transgenerational trauma is transmitted through relational processes that influence how individuals learn to regulate emotions, establish trust, construct identity, and navigate interpersonal relationships. Trauma, therefore, is not only remembered cognitively or carried biologically; it is also experienced and reproduced within the emotional bonds that connect one generation to the next.

#### 1.3.4. Family Systems Approaches

While attachment theory primarily focuses on dyadic relationships between caregivers and children, family systems approaches broaden the lens by conceptualizing trauma as a phenomenon that unfolds within interconnected relational networks. From this perspective, trauma is not viewed solely as an individual experience or psychological symptom but as a dynamic process that becomes embedded within the emotional, communicational, and organizational patterns of family life. The family itself becomes the primary unit of analysis, with traumatic experiences understood as influencing, and being maintained by, the interactions among its members across generations.

One of the foundational contributions to this perspective was provided by Abrams (1999), who argued that traumatic experiences often become woven into family structures, communication styles, emotional roles, and intergenerational expectations. Trauma, therefore, does not remain confined to the individual who originally experienced it. Instead, it becomes incorporated into the functioning of the family system, shaping how emotions are expressed, conflicts are managed, boundaries are negotiated, and relationships are maintained. Through these processes, trauma can persist even when the original traumatic event is no longer openly discussed or consciously remembered.

The integration of attachment and family systems perspectives further enriched this understanding. P. C. Alexander and Warner (2013) demonstrated that patterns of family violence and trauma are often sustained through enduring relational structures that influence multiple generations. Their work suggests that attachment dynamics and systemic family processes are deeply intertwined, jointly contributing to the reproduction of vulnerability and relational dysfunction over time.

Empirical research has provided substantial support for these theoretical assumptions. Fitzgerald et al. (2020) found that family systems variables contribute significantly to the transmission of trauma above and beyond the effects of individual psychopathology. Their findings suggest that understanding trauma transmission requires attention not only to the psychological functioning of individual family members but also to the quality of family relationships and patterns of interaction. Similarly, Bachem et al. (2018) demonstrated that parental posttraumatic stress symptoms, marital adjustment, and dyadic communication processes collectively influence offspring vulnerability, highlighting the importance of relational dynamics as mediators of trauma transmission.

From a systems-oriented perspective, Papero (2017) conceptualizes trauma as a multigenerational emotional process that reverberates throughout entire family systems. Rather than being transmitted in a linear fashion from one person to another, trauma influences the emotional climate of the family, shaping patterns of closeness, conflict, anxiety, and adaptation across generations. Similarly, Goff et al. (2020) emphasized the reciprocal relationship between posttraumatic symptoms and family functioning, arguing that trauma both influences and is influenced by ongoing relational processes.

More recent developments have expanded family systems theories beyond the boundaries of the family itself. Kelley et al. (2022) propose a socioecological family systems perspective, arguing that family functioning cannot be understood independently of the broader social environments in which families are embedded. Economic conditions, cultural norms, community resources, and societal stressors all influence how trauma is experienced, interpreted, and transmitted. Extending this systems-oriented perspective, Eads (2023) further emphasizes the value of integrating social constructivism and systems theory to understand how young people and families navigate posttrauma realities, highlighting the role of relational and socially constructed meanings in processes of adaptation following trauma. Economic conditions, cultural norms, community resources, and societal stressors all influence how trauma is experienced, interpreted, and transmitted. This broader ecological perspective acknowledges that family systems operate within larger systems that may either exacerbate or mitigate trauma-related vulnerabilities.

Eads (2023) further advances this view through the integration of social constructivism and systems theory. According to this perspective, posttraumatic realities are not simply inherited but continuously co-constructed through interactions among individuals, families, communities, and social institutions. Trauma becomes part of an evolving network of meanings and relationships, shaped by the ways people collectively interpret and respond to past experiences.

A similar emphasis on hidden relational processes can be found in the work of Minulescu (2016), who, drawing on analytical psychotherapy, argues that transgenerational trauma frequently manifests through unconscious family narratives, symbolic legacies, and repetitive relational configurations. More recently, Bowe et al. (2025) have integrated multiple theoretical perspectives on intergenerational trauma, violence, and maltreatment, emphasizing their implications for therapeutic responses and reinforcing the need for multidimensional frameworks capable of connecting theoretical explanations with clinical practice. These invisible psychological inheritances influence identity formation and interpersonal functioning, often operating outside conscious awareness while continuing to shape the developmental experiences of subsequent generations.

Taken together, family systems approaches have significantly expanded the understanding of transgenerational trauma by demonstrating that traumatic experiences are not transmitted solely through biology or individual psychology. Instead, they are embedded within relational systems that organize emotions, meanings, expectations, and patterns of interaction across generations. Reflecting the growing consensus within the field, Perry et al. (1995) conclude that contemporary theories of intergenerational trauma increasingly require the integration of biological, psychological, relational, and ecological perspectives. Such integration is essential for capturing the complexity of trauma transmission and for developing interventions capable of addressing not only individual symptoms but also the broader systems within which trauma is maintained and reproduced.

This growing theoretical diversity has led several authors to argue that no single framework is currently capable of explaining the complexity of intergenerational trauma across biological, relational, developmental, and sociocultural levels. Instead, recent integrative perspectives emphasize the need to combine multiple explanatory traditions in order to better understand the persistence of trauma-related vulnerability and to inform therapeutic interventions (Bowe et al., 2025). This emerging consensus provides an important rationale for the development of broader integrative frameworks such as the Contextual Resonance Theory of Psychopathology.

### 1.4. Conceptual Limitations of Existing Models

Despite the substantial progress achieved by contemporary trauma research, important conceptual gaps remain. Existing frameworks have significantly advanced our understanding of how traumatic experiences may affect subsequent generations, yet each tends to emphasize a particular level of analysis while providing only a partial explanation of the broader phenomenon. Genetic and epigenetic models primarily focus on biological mechanisms and inherited vulnerabilities. Attachment-based approaches concentrate on early relational experiences and the transmission of internal working models. Family systems theories emphasize interpersonal dynamics and multigenerational emotional processes, whereas historical, cultural, and bioecological perspectives direct attention toward collective memory, sociocultural continuity, and community-level influences.

Collectively, these approaches have enriched the field and generated valuable empirical evidence. However, they leave a fundamental question only partially answered: how do the consequences of trauma continue to shape psychological functioning long after the original traumatic event has ended, the original perpetrators have disappeared, and, in some cases, even the conscious memory of the trauma has faded?

Recent evidence continues to support the existence of transgenerational trauma while simultaneously highlighting the considerable heterogeneity of the underlying mechanisms. A recent systematic review and meta-analysis by Zasiekina et al. (2026) concluded that although transgenerational effects are consistently observed across populations exposed to genocide and mass violence, substantial variability remains regarding the relative contribution of biological, psychological, relational, and contextual pathways. These findings reinforce the need for integrative theoretical models capable of explaining how multiple mechanisms interact rather than privileging a single explanatory level.

A closer examination of the literature reveals that many existing theories implicitly assume that trauma is transmitted from one person to another through biological inheritance, caregiving relationships, family interactions, or cultural narratives. Yet the evidence reviewed across these perspectives points toward a potentially broader and largely unexplored possibility. What may persist across generations is not trauma itself, but the contexts that trauma creates. Individuals may transmit more than symptoms, memories, or relational patterns; they may reproduce environments that have already been shaped by traumatic adaptation.

Within such environments, children do not merely encounter traumatized caregivers. They grow up in worlds organized around hypervigilance, emotional restraint, distrust, unpredictability, rigid control, chronic threat anticipation, or persistent uncertainty. Over time, these characteristics become normalized features of everyday life. They are woven into communication styles, family rules, institutional practices, social expectations, and collective assumptions about safety, trust, and belonging. Importantly, these contextual characteristics may endure independently of explicit trauma narratives, conscious recollection, or even direct knowledge of the original traumatic experience.

This observation exposes a significant limitation of current models. While they successfully explain how trauma influences individuals, relationships, families, or communities, they provide less clarity regarding how trauma-related adaptations become embedded within environments themselves and continue to exert psychological influence across generations. Existing theories can explain why trauma survivors remain affected by their experiences; they are less equipped to explain why entire social systems, including families, organizations, institutions, and communities, often continue to reproduce trauma-related patterns long after the initiating event has disappeared.

As a result, contemporary models struggle to fully account for the remarkable persistence of psychological vulnerability across time and context. The endurance of trauma-related adaptations suggests that transmission may involve more than biological inheritance, attachment processes, family dynamics, or cultural memory alone. It raises the possibility that environments themselves may become carriers of traumatic adaptations, continuously shaping the expectations, perceptions, and emotional responses of those who inhabit them. Understanding how such trauma-shaped contexts emerge, persist, and influence mental health across generations represents a critical theoretical challenge that remains insufficiently addressed within existing frameworks.

### 1.5. Aim of the Paper

Building upon these conceptual limitations, the present paper introduces the Contextual Resonance Theory of Psychopathology (CRTP), a cognitive–systemic framework designed to explain how trauma-related vulnerability persists through the recursive organization of trauma-shaped contexts across generations. Drawing on insights from historical trauma research, attachment theory, family systems approaches, socioecological models, predictive processing, and contemporary trauma scholarship, the proposed framework advances a central proposition: trauma is maintained across generations not primarily through the direct transmission of symptoms, memories, or biological alterations, but through the recursive reproduction of contexts that have themselves been shaped by traumatic adaptation. The purpose of CRTP is therefore not to replace existing theories of trauma transmission, but to provide an integrative explanatory architecture capable of describing how mechanisms identified by different theoretical traditions interact across biological, cognitive, relational, institutional, and sociocultural levels.

From this perspective, the enduring effects of trauma reside not only within individuals but also within the relational, familial, organizational, and cultural environments they create and inhabit. These environments gradually acquire characteristic patterns of functioning, such as hypervigilance, distrust, emotional restriction, chronic uncertainty, rigid control, or threat anticipation, which become normalized and repeatedly transmitted to subsequent generations. As a result, descendants may inherit not the trauma itself, but the conditions through which trauma continues to organize perception, behavior, and social interaction.

To explain these processes, the theory introduces three interconnected concepts: contextual traumatic inertia, recursive contextual resonance, and distributed psychopathology. Together, these constructs provide a framework for understanding how trauma-related adaptations become embedded within environments, how such environments continually reinforce particular expectations about safety and danger, and how psychological vulnerability may emerge from ongoing interactions between individuals and trauma-shaped contexts. Within this model, clinically significant psychopathology is no longer conceptualized exclusively as an individual condition but as an emergent phenomenon arising from the dynamic interaction between individuals and trauma-shaped environments. This formulation does not imply that all trauma-related adaptations are psychopathological. Rather, adaptive responses become clinically relevant only when they persist beyond their original adaptive context and contribute to clinically significant distress, functional impairment, or substantial disruption of psychological, interpersonal, occupational, or social functioning.

In addition to offering a new explanatory framework for transgenerational trauma, the paper proposes corrective recontextualization as a complementary mechanism of recovery. Rather than viewing healing solely as an intrapsychic process, corrective recontextualization emphasizes the transformative potential of sustained exposure to psychologically restorative environments capable of challenging and gradually recalibrating maladaptive predictive models of threat, trust, belonging, and safety. Recovery, therefore, is understood not only as a change within the individual but also as a change in the contexts through which experience is continuously interpreted and regulated.

By shifting the unit of analysis from the isolated individual to the recursive interaction between individuals and their environments, the Contextual Resonance Theory of Psychopathology seeks to provide an integrative theoretical foundation for future research on trauma, resilience, psychopathology, and mental health across generations. More broadly, it aims to contribute to a growing body of scholarship that recognizes psychological functioning as inseparable from the contexts in which it is constructed, maintained, and transformed.

Although CRTP shares several assumptions with ecological, developmental, and systems-oriented theories, its principal value lies in generating a set of theoretically distinctive predictions that emerge specifically from the concept of trauma-shaped contextual organization. These predictions extend beyond the general proposition that contexts influence psychological functioning by specifying how contextual systems themselves acquire adaptive continuity, preserve trauma-related organization, and interact recursively with successive generations. Unlike existing ecological or transactional frameworks, which primarily predict reciprocal influences between individuals and environments, CRTP predicts that trauma-shaped contextual organization may persist independently of the original traumatic event and continue influencing individuals who possess no direct biological or familial connection to the initiating trauma. This proposition represents one of the central empirical predictions through which CRTP can be distinguished from related theoretical approaches.

### 1.6. Theoretical Integration Underlying the Contextual Resonance Theory of Psychopathology

The Contextual Resonance Theory of Psychopathology (CRTP) is not intended to replace existing theories of transgenerational trauma but rather to integrate their complementary explanatory contributions within a unified cognitive–systemic framework. The development of CRTP was motivated by the observation that current theoretical traditions have significantly advanced the understanding of trauma transmission, yet each primarily explains specific dimensions of the phenomenon while leaving important questions unresolved. Biological models emphasize inherited vulnerability, attachment theory focuses on early relational representations, family systems approaches describe recursive interpersonal dynamics, ecological models highlight environmental influences, predictive processing explains cognitive adaptation, and resilience research identifies protective mechanisms. However, no single perspective simultaneously accounts for the continuous interactions among biological, cognitive, relational, institutional, and sociocultural processes that collectively shape the persistence or interruption of transgenerational trauma.

Rather than viewing these perspectives as competing explanations, CRTP conceptualizes them as complementary levels of analysis operating within a recursive adaptive system. Biological mechanisms influence cognitive expectations; cognitive schemas shape interpersonal relationships; relational dynamics become embedded within families, institutions, and communities; and these broader contexts continuously reinforce or modify individual predictive models. Consequently, trauma transmission is understood not as the product of a single causal pathway but as the emergent consequence of reciprocal interactions occurring across multiple systemic levels.

Importantly, CRTP does not assume that the theoretical traditions it integrates are interchangeable or fully equivalent. Rather, they operate at complementary levels of analysis within a multilevel explanatory architecture. Predictive processing primarily explains how individuals construct and continuously update expectations about the world through predictive inference, whereas attachment theory explains how many of these predictive models are initially shaped within early caregiving relationships. Similarly, neurodevelopmental perspectives describe the biological embedding of adaptation, while ecological and socioecological models explain how environmental conditions continuously reinforce, modify, or recalibrate these biological and cognitive processes throughout development. Family systems approaches primarily address recurrent interactional patterns within relational systems, whereas cultural and sociological perspectives explain how these patterns become embedded within broader institutional, historical, and societal contexts. Rather than viewing these perspectives as competing explanations, CRTP conceptualizes them as interacting mechanisms operating across different but mutually influential levels of organization, thereby providing an integrative framework linking biological, cognitive, relational, contextual, and sociocultural processes.

The recursive organization proposed by CRTP also shares important conceptual affinities with Giddens’ Structuration Theory, which conceptualizes social structures as both the medium and the outcome of recurrent social practices (Giddens, 1984). From this perspective, individuals continuously reproduce or transform the institutional and relational contexts within which they act, while those same contexts simultaneously shape future action. CRTP adopts this recursive understanding of person–environment relations but extends it by proposing that trauma-shaped contexts may progressively acquire adaptive continuity, allowing patterns originally developed in response to adversity to persist independently of the initiating traumatic event. In this framework, recursive contextual organization is sustained not only through institutional arrangements but also through repeated interaction rituals embedded in families, peer networks, educational settings, workplaces, and communities (Collins, 2004). Such everyday relational practices continuously reinforce expectations regarding safety, trust, threat, belonging, and emotional regulation, thereby contributing to the maintenance or, alternatively, the transformation of trauma-related contextual organization across generations. Consequently, CRTP conceptualizes recursive interaction not merely as the reproduction of social structure, but as the mechanism through which adaptive contextual systems evolve, stabilize, and become potential targets for therapeutic and societal intervention.

A central contribution of CRTP is therefore the proposition that psychopathology cannot be adequately understood solely as an individual characteristic or inherited vulnerability. Instead, psychopathological processes emerge through continuous resonance between individuals and the contexts in which they repeatedly operate. In this perspective, contexts function not merely as external influences but as active systems capable of maintaining, amplifying, or transforming trauma-related predictive structures across generations. Consequently, therapeutic intervention extends beyond symptom reduction toward the deliberate modification of relational, institutional, and sociocultural environments capable of supporting adaptive cognitive restructuring through processes of Corrective Recontextualization (Table 1).

Importantly, this integrative perspective should not be interpreted as a reformulation of existing transactional or ecological models. Rather, CRTP extends these traditions by conceptualizing trauma-shaped contexts as adaptive systems possessing organizational continuity across generations. In doing so, the theory shifts the explanatory focus from reciprocal interaction alone to the persistence, inheritance, and transformation of contextual organization itself.

### 1.7. Positioning CRTP Within Existing Integrative Theories

Several existing theoretical traditions have proposed reciprocal interactions between individuals and their environments. Bandura’s reciprocal determinism conceptualizes dynamic relationships among personal factors, behaviour, and environmental influences. Sameroff’s transactional model similarly describes development as a continuous process of mutual influence between children and their caregiving environments. Niche Construction Theory further demonstrates that organisms actively modify their environments and subsequently inherit the ecological consequences of those modifications across generations.

CRTP builds upon these important theoretical traditions rather than contradicting them. However, its primary contribution lies elsewhere. Whereas these models conceptualize environments primarily as dynamic interaction partners, developmental contexts, or ecological products of organism–environment reciprocity, CRTP proposes that contexts themselves may progressively acquire trauma-related organizational properties that function as active carriers of adaptive information across generations. In this perspective, contexts are not merely influences on psychological functioning but become adaptive systems capable of preserving, reproducing, amplifying, or transforming trauma-related predictive structures independently of the original traumatic event.

Consequently, the principal theoretical contribution of CRTP is not the introduction of reciprocity itself, which is already well established in developmental, ecological, and systems-oriented psychology, but the reconceptualization of trauma-shaped contexts as autonomous systemic units of adaptive continuity. Rather than treating contexts merely as environmental influences or interaction partners, CRTP conceptualizes them as dynamic systems capable of preserving, reproducing, amplifying, and transforming trauma-related adaptive organization across generations. This shift changes the primary unit of analysis from individual–environment interaction toward recursive person–context systems, thereby providing an integrative explanatory architecture capable of connecting biological, cognitive, relational, institutional, and sociocultural mechanisms within a common multilevel framework.

A comparative overview of CRTP and related theoretical frameworks is presented in Table 2.

## 2. From Individual Trauma to Contextual Trauma

### 2.1. Trauma as Environmental Adaptation

Traditional psychopathological models have frequently conceptualized trauma as a disruptive event that produces dysfunction within the individual. From this perspective, symptoms such as hypervigilance, emotional dysregulation, avoidance, dissociation, or persistent threat anticipation are often interpreted as indicators of impairment resulting from exposure to overwhelming experiences. However, a growing body of research suggests that such responses may be more accurately understood as adaptive mechanisms that emerge in response to dangerous, unpredictable, or chronically stressful environments. Rather than representing failures of psychological functioning, many trauma-related reactions appear to reflect attempts by the individual to maintain safety, predictability, and survival under adverse conditions.

To avoid conceptual ambiguity, the term context is used throughout this paper in a specific theoretical sense. Within the Contextual Resonance Theory of Psychopathology (CRTP), context does not simply refer to the external environment or to any circumstance surrounding an individual. Rather, context is conceptualized as a dynamic, multilevel system of interacting biological, cognitive, relational, institutional, cultural, and historical conditions that continuously shape, reinforce, and modify predictive models of reality and patterns of adaptive behavior. These contextual levels are not viewed as independent influences but as reciprocally interacting components of a single adaptive system operating across time.

Within CRTP, context encompasses interacting biological, cognitive, relational, institutional, cultural, and historical processes. However, not every environmental influence constitutes a theoretically relevant context. A context becomes analytically meaningful only when it participates in recursive interactions that maintain, amplify, or transform trauma-related predictive organization over time.

This adaptive perspective has deep roots in developmental, evolutionary, ecological, and resilience-oriented approaches to trauma. One of the most influential formulations was proposed by Perry and Pollard (1998), who argued that the developing brain organizes itself around repeated patterns of experience through a process of use-dependent neurodevelopment. According to their model, neural systems become progressively specialized in response to environmental demands. When children grow up in environments characterized by threat, instability, or chronic stress, the brain adapts accordingly, prioritizing rapid threat detection, physiological arousal, and survival-oriented responding. Over time, transient adaptive states become enduring traits, creating patterns of functioning that may appear maladaptive in safe environments but remain highly functional within dangerous contexts.

Expanding this perspective, trauma has been described as a challenge to homeostasis that activates a cascade of neurobiological adaptations aimed at preserving survival (Benight, 2012). Stress-response systems, including autonomic, endocrine, emotional, and cognitive processes, become recalibrated in response to environmental conditions. Importantly, these adaptations should not be viewed as pathological in themselves. Rather, they represent biologically sensible responses to environments in which danger is frequent, unpredictable, or unavoidable. What later appears as psychopathology may initially have been a highly effective survival strategy (Benight, 2012).

A similar perspective has emerged within resilience research, where adaptation to traumatic stress is conceptualized as a dynamic process through which individuals continuously attempt to restore efficacy, control, and coherence in the face of adversity rather than merely as a constellation of pathological symptoms (Konner, 2007). From this perspective, the critical question is not why trauma produces symptoms, but how individuals actively adapt to environmental demands and how these adaptations shape future functioning (Konner, 2007).

The adaptive nature of trauma responses is further supported by evolutionary perspectives, which suggest that many psychological responses associated with trauma—including heightened vigilance, increased sensitivity to threat, rapid emotional reactivity, and social caution—may have evolved because they enhanced survival under dangerous environmental conditions (Harvey, 1996). Although these responses can become costly when environmental threats subside, they remain fundamentally adaptive when viewed within the contexts in which they originally emerged. Trauma-related behaviors therefore reflect not merely psychological injury but evolutionary strategies designed to maximize survival in hostile environments.

Ecological models further reinforce the importance of context in understanding adaptation. Ungar (2013) argued that trauma cannot be adequately understood by focusing exclusively on individual symptoms because recovery and adaptation are deeply influenced by the ecological systems surrounding the person. Family relationships, social networks, cultural resources, institutional supports, and community environments all contribute to shaping how traumatic experiences are interpreted and managed. Trauma therefore represents an ongoing interaction between individuals and the environments in which they are embedded rather than a purely intrapsychic phenomenon.

The role of context becomes even more apparent when considering resilience following trauma. Ellis et al. (2012) challenged individualistic conceptualizations of resilience by arguing that adaptation depends not only on personal characteristics but also on access to supportive social, cultural, and environmental resources. Resilience emerges when environments provide opportunities for safety, belonging, meaning, and support. Consequently, both vulnerability and adaptation must be understood as products of person–context interactions rather than as characteristics residing solely within individuals.

Research on trauma across generations similarly supports an adaptive interpretation of trauma-related processes. Descendants of trauma survivors inherit not only vulnerabilities but also capacities for resilience, adaptation, and survival (R. D. Goodman, 2013). Their work suggests that many characteristics commonly interpreted as indicators of pathology may originally have emerged as adaptive responses to environmental demands experienced by previous generations.

At the population level, evidence also indicates that environmental experiences shape behavioral orientations far beyond the immediate trauma context. Ellis et al. (2012), Lange et al. (2022), Raja and Carrico (2022), and Stein et al. (2002) found that formative adverse experiences influence later patterns of environmental engagement, demonstrating how early environmental conditions continue to shape perceptions, priorities, and behavioral responses throughout the lifespan. Similarly, Keller and Mrsic-Flogel (2018) showed that exposure to traumatic environments interacts with genetic predispositions, reinforcing the conclusion that vulnerability emerges from the dynamic interplay between biology and context rather than from either factor alone.

Perhaps the most direct evidence for the importance of environmental adaptation comes from intervention research. Lange et al. (2022), evaluating Trauma Systems Therapy, demonstrated that meaningful improvements in trauma-related outcomes often require modifications not only at the individual level but also within the environments in which individuals live. Their findings suggest that psychological adaptation cannot be separated from environmental conditions and that lasting recovery frequently depends upon changes in contextual factors. This conclusion is echoed by Clark (2015), Keller and Mrsic-Flogel (2018), Raja and Carrico (2022), and Stein et al. (2002), whose work further highlights the importance of developmental, environmental, and contextual processes in shaping adaptation.

### 2.2. The Emergence of Trauma-Shaped Contexts

If trauma-related responses are understood as adaptive reactions to environmental demands, an important question immediately follows: what happens when these adaptations persist after the original threat has disappeared? Existing trauma theories have largely focused on how individuals adapt to adverse environments. Far less attention has been devoted to the reciprocal process through which environments themselves become reorganized around traumatic adaptations. Yet many of the studies reviewed in the previous section implicitly suggest that trauma does not remain confined to the individual. Rather, it gradually reshapes the relational, familial, social, and cultural contexts in which future generations develop.

From a neurodevelopmental perspective, Clark (2015) argued that repeated exposure to threat produces predictable adaptations in cognition, emotion, and behavior. Individuals become more vigilant, more cautious, more sensitive to uncertainty, and more likely to prioritize safety over exploration. While these adaptations initially serve protective functions, they also influence how people interact with others, raise children, form relationships, establish social norms, and construct institutions. Over time, adaptive responses to trauma may become embedded within the environments individuals create around themselves.

This process can be understood as a form of contextual stabilization. What begins as an individual adaptation gradually acquires environmental expression. Hypervigilant individuals often create highly controlled environments. Individuals who have learned that trust is dangerous may communicate caution and suspicion to others. Families shaped by traumatic experiences may organize themselves around emotional restraint, avoidance of vulnerability, rigid role expectations, or excessive monitoring of potential threats. These patterns eventually become part of the context itself rather than merely characteristics of particular individuals.

Contemporary philosophical perspectives further extend this interpretation by suggesting that traumatic experience may persist not only through explicit memories but also through embodied, relational, and mimetic processes embedded within social environments. Transgenerational trauma may be sustained through shared bodily practices, relational patterns, and vicarious memories that transcend direct autobiographical recollection. This perspective is consistent with the present framework, which proposes that contexts themselves become carriers of trauma-shaped adaptation.

The role of context becomes even more apparent when considering resilience following trauma. Ellis et al. (2012) challenged individualistic conceptualizations of resilience by arguing that adaptation depends not only on personal characteristics but also on access to supportive social, cultural, and environmental resources. Resilience emerges not solely from personal strengths but from access to social and contextual resources that support adaptation. By implication, environments characterized by chronic threat, instability, exclusion, or mistrust may also become mechanisms through which vulnerability is maintained. The same contextual processes that support resilience under favorable conditions may support the persistence of trauma under adverse conditions.

Evidence from intergenerational trauma research reinforces this observation. R. D. Goodman (2013) argues that descendants of trauma survivors inherit more than biological or psychological vulnerabilities. They also inherit patterns of coping, expectations about safety and danger, family narratives, and behavioral strategies developed in response to earlier adversities. These inherited adaptations contribute to the construction of environments that continue to reflect the logic of the original trauma, even when direct exposure has ceased.

A similar dynamic emerges from studies of collective and historical trauma. Communities exposed to prolonged violence, displacement, persecution, or systemic oppression often develop social norms and cultural practices designed to reduce future vulnerability. Although such adaptations may initially serve protective purposes, they can persist long after the original danger has disappeared. Collective memories of threat may continue to shape educational practices, parenting styles, institutional structures, and community relationships. Over time, trauma becomes woven into the organizational principles of everyday life.

The importance of environmental conditions is further demonstrated by intervention research. Lange et al. (2022) found that effective trauma treatment frequently requires modifications to the individual’s surroundings rather than focusing exclusively on symptom reduction. Improvements in functioning were closely linked to changes in environmental stability, relational safety, and contextual support. Similarly, Raja and Carrico (2022) observed that successful trauma-focused interventions often depend upon adaptation to the specific social and environmental realities of those receiving treatment. These findings suggest that trauma-related difficulties cannot be fully understood independently of the contexts in which they occur.

Importantly, trauma-shaped contexts need not be overtly traumatic. They often emerge through subtle and cumulative processes. An environment organized around chronic vigilance may appear responsible and protective. Emotional restraint may be interpreted as maturity. Distrust may be framed as wisdom. Excessive control may be justified as preparedness. Consequently, trauma-shaped adaptations can become normalized and institutionalized, losing their apparent connection to the original traumatic event while continuing to influence behavior and development.

This observation points to a critical limitation of conventional trauma frameworks. Most models conceptualize trauma as something carried by individuals, families, or groups. However, an equally plausible interpretation is that trauma gradually becomes embedded within contexts themselves. The family, school, workplace, community, or institution begins to function according to assumptions established by earlier experiences of threat. These assumptions shape expectations about trust, safety, belonging, autonomy, vulnerability, and control for those who enter the system, including individuals with no direct connection to the original trauma.

From this perspective, transgenerational trauma is not simply the transmission of symptoms, memories, or biological alterations. It is also the reproduction of environments organized around adaptations to past adversity. Such environments continuously communicate information about danger and safety, influencing the predictive models through which individuals interpret reality. Trauma therefore persists not only because people remember it, but because the contexts shaped by trauma continue to exist and continue to teach.

### 2.3. Why Psychopathology Cannot Be Understood Exclusively at the Individual Level

The recognition that trauma-related adaptations may become embedded within environments challenges one of the most enduring assumptions in psychology and psychiatry: the notion that psychopathology resides primarily within the individual. Although contemporary mental health research increasingly acknowledges the importance of social, relational, and cultural influences, most diagnostic systems continue to conceptualize psychological disorders as characteristics of persons rather than as products of ongoing interactions between persons and contexts. Symptoms are measured at the individual level, diagnoses are assigned to individuals, and interventions are frequently directed toward modifying individual cognition, emotion, or behavior. While this approach has generated important advances, it may overlook fundamental contextual processes involved in the development and maintenance of psychological suffering.

From an ecological perspective, psychological functioning cannot be separated from the environments in which it emerges. Ungar (2013) argued that trauma recovery and adaptation are embedded within multiple interacting systems that extend far beyond the individual. Family relationships, community resources, institutional structures, cultural narratives, and broader social conditions continuously influence psychological outcomes. Consequently, what appears to be an individual symptom may often reflect a broader adaptation to environmental realities.

This observation is particularly evident in developmental trauma research. Clark (2015) and Walsh et al. (2020) demonstrated that many trauma-related symptoms emerge through repeated exposure to specific environmental conditions during critical developmental periods. Hyperarousal, emotional dysregulation, attentional difficulties, and heightened threat sensitivity are not random dysfunctions but predictable adaptations to contexts characterized by chronic stress and uncertainty. Once established, these adaptations continue to influence behavior even when environmental conditions change. However, their origins remain fundamentally relational and contextual rather than exclusively individual.

Swanson (2016) similarly challenged the tendency to locate pathology solely within the person. He argued that adaptation following trauma is a dynamic process involving continuous interactions between individuals and their environments. From this perspective, psychological difficulties should not be understood merely as deficits or disorders but as attempts to maintain functioning under adverse conditions. What is labeled as psychopathology may therefore represent the persistence of adaptive strategies that have become mismatched to current environmental demands.

Evolutionary perspectives further support this argument. Williams (2018) observed that many trauma-related responses possess clear adaptive value within dangerous environments. Hypervigilance, heightened emotional sensitivity, social caution, and rapid threat detection may increase survival in contexts where danger is frequent and unpredictable. These same characteristics may become problematic in environments that no longer require such adaptations. The apparent pathology thus emerges not solely from the individual response but from the relationship between that response and the surrounding environment.

Evidence from resilience research also challenges purely individualistic explanations. Ellis et al. (2012) argue that resilience is not a personal trait residing within individuals but a process emerging from successful negotiations between individuals and their environments. Access to supportive relationships, social inclusion, cultural resources, and opportunities for meaningful participation often predicts adaptation more effectively than individual characteristics alone. If resilience cannot be understood independently of context, vulnerability may likewise depend upon contextual conditions rather than solely upon personal deficits.

Research on trauma across generations further complicates individual-centered models. R. D. Goodman (2013) demonstrates that descendants of trauma survivors frequently develop within environments shaped by previous adaptations to threat. Their vulnerabilities cannot be fully explained by genetic inheritance, attachment patterns, or family dynamics alone because these factors are themselves embedded within broader contextual systems. The environments inherited by subsequent generations often contain implicit assumptions about danger, trust, uncertainty, and control that continue to shape development regardless of whether the original trauma remains consciously remembered.

Intervention research provides some of the strongest evidence against exclusively individual explanations of psychopathology. Lange et al. (2022), evaluating Trauma Systems Therapy, found that sustainable improvements often required environmental modifications alongside individual treatment. Changes in family stability, school functioning, community support, and relational safety were essential components of recovery. Similarly, Raja and Carrico (2022) reported that effective trauma-focused interventions frequently depend upon adaptation to contextual realities rather than the uniform application of symptom-focused techniques. These findings suggest that addressing individual symptoms without addressing environmental conditions may leave important determinants of psychological functioning unchanged.

The importance of context is also evident in research examining the long-term consequences of adversity. Stein et al. (2002) found that childhood trauma and formative life experiences influence not only psychological outcomes but also broader patterns of engagement with social and environmental systems. Such findings indicate that trauma shapes how individuals perceive, interpret, and interact with the world around them, further blurring the distinction between internal psychological processes and external environmental conditions.

Even studies emphasizing biological vulnerability point toward the inseparability of person and context. Keller and Mrsic-Flogel (2018) demonstrated that genetic influences on posttraumatic stress symptoms operate in conjunction with environmental exposures. Vulnerability does not emerge from biology alone but from the interaction between biological predispositions and lived experiences. This interactional pattern appears consistently across levels of analysis, suggesting that psychopathology may be more accurately conceptualized as a property of person–context systems rather than as an attribute of isolated individuals.

Taken together, these findings support a fundamental theoretical shift. Rather than viewing psychopathology as something located exclusively within the individual, it may be more useful to understand it as an emergent phenomenon arising from ongoing interactions between individuals and the environments they inhabit. Symptoms do not occur in isolation; they develop within relational, social, institutional, and cultural contexts that continuously shape perception, emotion, behavior, and meaning-making. In many cases, psychological suffering may reflect not only individual dysfunction but also the persistence of contexts organized around threat, instability, exclusion, unpredictability, or chronic stress.

## 3. The Contextual Resonance Theory of Psychopathology

The Contextual Resonance Theory of Psychopathology (CRTP) is proposed as an integrative framework addressing this gap. The theory builds upon ecological approaches to trauma (Eads, 2023), neurodevelopmental adaptation models (Benight, 2012; Perry & Pollard, 1998), resilience research (Goff et al., 2020; Minulescu, 2016), multigenerational trauma frameworks (R. D. Goodman, 2013; Starrs & Békés, 2025), attachment-based models (Ghai & Kader, 2022; S. Goodman, 2024), and family systems perspectives (Bachem et al., 2018; Fitzgerald et al., 2020; Papero, 2017). However, it introduces a fundamental shift in the unit of analysis. Rather than conceptualizing trauma transmission primarily as a process occurring between individuals, the theory proposes that trauma persists through the recursive reproduction of trauma-shaped contexts.

According to CRTP, psychopathology emerges not exclusively from individual vulnerability, but from ongoing interactions between individuals and environments that continue to embody adaptations originally developed in response to adversity. Trauma survives because contexts survive.

### 3.1. Core Assumptions of the Theory

Within CRTP, three analytically distinct levels are distinguished.

- Adaptive contextual calibration refers to functional cognitive, emotional and behavioural responses that increase adaptation within threatening environments.
- Enduring vulnerability refers to the persistence of trauma-shaped predictive expectations after the original threat has disappeared, even in the absence of clinically significant distress or impairment.
- Clinically significant psychopathology refers only to situations in which these persistent adaptations become inflexible, contextually maladaptive, and are accompanied by clinically significant distress, functional impairment, or substantial disruption of psychological, interpersonal, occupational, or social functioning.

The Contextual Resonance Theory of Psychopathology rests upon five foundational assumptions.

First, trauma-related responses are fundamentally adaptive. Consistent with neurodevelopmental and evolutionary perspectives, behaviors commonly classified as symptoms, including hypervigilance, emotional withdrawal, distrust, avoidance, and threat anticipation, are initially functional responses to dangerous environments (Benight, 2012; Harvey, 1996; Konner, 2007; Perry & Pollard, 1998). These adaptations emerge because they increase the probability of survival under adverse conditions.

Second, adaptive responses do not remain confined to individuals. Over time, they influence family interactions, social norms, institutional practices, and cultural expectations. What begins as a personal adaptation gradually acquires contextual expression. Trauma therefore modifies not only persons but also the environments they create and inhabit.

Third, contexts possess memory. Although environments do not remember in a biological sense, they preserve adaptations through routines, expectations, communication styles, organizational structures, and shared assumptions. Families, communities, schools, and institutions often continue operating according to rules established during previous periods of threat long after the original danger has disappeared.

Fourth, individuals continuously learn from the contexts they inhabit. Drawing from predictive processing principles, people construct models of reality based on recurring environmental regularities. Contexts characterized by distrust, unpredictability, exclusion, or chronic vigilance repeatedly communicate information regarding safety and danger. Over time, these contextual signals shape expectations about the world and influence future adaptation.

Finally, psychopathology should be understood as an emergent property of person–context interactions rather than as an exclusively individual phenomenon. Psychological symptoms reflect not only internal dysfunction but also ongoing adaptation to environmental conditions. Consequently, the persistence of psychopathology often reflects the persistence of trauma-shaped contexts.

These assumptions provide the conceptual foundation for three novel constructs: contextual traumatic inertia, recursive contextual resonance, and distributed psychopathology.

### 3.2. Contextual Traumatic Inertia

The first core construct introduced by CRTP is contextual traumatic inertia.

Contextual traumatic inertia refers to the tendency of social, relational, and institutional systems to continue reproducing adaptations originally developed in response to traumatic conditions even after those conditions no longer exist.

The concept draws inspiration from ecological and systems perspectives suggesting that once systems become organized around particular patterns of functioning, they tend to preserve those patterns over time (Ungar, 2013). In the context of trauma, adaptations that were once protective may become normalized and subsequently embedded within everyday practices.

For example, a family that survived prolonged periods of instability may continue emphasizing vigilance and control long after stability has been restored. A community shaped by collective violence may maintain patterns of distrust despite the absence of immediate threats. Organizations formed under conditions of uncertainty may preserve rigid hierarchies and defensive decision-making structures even when environmental demands have fundamentally changed.

Importantly, contextual traumatic inertia does not require conscious awareness of the original trauma. The initiating event may be forgotten, minimized, or unknown to subsequent generations. What persists is not necessarily the memory of trauma but the organizational logic created by trauma.

As a result, vulnerability may persist independently of biological inheritance, explicit family narratives, or direct exposure to adversity because the contextual conditions that originally generated adaptive responses remain active.

From an empirical perspective, Contextual Traumatic Inertia may be operationalized as the persistence of trauma-shaped contextual characteristics across time despite the absence of the original traumatic conditions. Potential indicators include stable family communication patterns, institutional rigidity, perceived environmental unpredictability, chronic threat anticipation, and persistent trauma-related cognitive schemas observed across successive generations.

These indicators may be assessed using a combination of standardized questionnaires, observational methods, and archival data. Family communication patterns and cohesion may be evaluated using validated family functioning instruments (e.g., the Family Environment Scale or FACES-IV), whereas institutional rigidity may be examined through organizational climate questionnaires, policy analyses, or longitudinal archival records documenting institutional practices over time. Perceived environmental unpredictability and chronic threat anticipation may be assessed using self-report measures of environmental uncertainty, perceived safety, and threat-related cognitive schemas. Combining these approaches across multiple generations would allow researchers to quantify the persistence of trauma-shaped contextual organization.

Longitudinal cohort studies, multilevel analyses, and cross-generational family designs would provide appropriate methodological approaches for examining the stability and transmission of these contextual characteristics. Consistent with CRTP, the theory would be challenged if trauma-shaped contextual patterns failed to demonstrate measurable persistence over time or showed no association with subsequent psychological adaptation.

CRTP is therefore not a general theory of all psychopathology but a theory explaining how context-mediated vulnerability may, under specific conditions, contribute to psychopathology.

### 3.3. Recursive Contextual Resonance

The second central construct is recursive contextual resonance.

Recursive contextual resonance describes the continuous feedback process through which trauma-shaped environments reinforce trauma-related expectations, which in turn contribute to the reproduction of trauma-shaped environments.

The concept builds upon ecological, developmental, and predictive perspectives emphasizing reciprocal interactions between individuals and contexts (Bachem et al., 2018; Eads, 2023; Goff et al., 2020; Kelley et al., 2022; Papero, 2017).

Individuals enter environments containing implicit assumptions regarding trust, danger, belonging, autonomy, and safety. Through repeated exposure, these assumptions become incorporated into cognitive and emotional functioning. Individuals subsequently behave in ways that reflect those learned expectations, thereby contributing to the maintenance of the very environments from which those expectations originated.

For example, an environment characterized by chronic distrust may foster suspicious interpretations of social interactions. These interpretations promote defensive behaviors, which subsequently reduce trust and reinforce the original contextual climate. Similarly, environments organized around threat anticipation may produce heightened vigilance among members, whose vigilance further contributes to perceptions of danger within the system.

Over time, the original source of trauma becomes increasingly irrelevant to the maintenance of the system. The resonance process itself becomes self-sustaining.

From this perspective, transgenerational trauma persists not because traumatic memories are directly transferred across generations, but because each generation encounters environments already organized around adaptations established by previous generations.

Recursive Contextual Resonance may be empirically examined through indicators capturing reciprocal interactions between individual expectations and contextual characteristics over time. Empirical assessment may combine repeated measurements of individual predictive expectations (e.g., threat-related beliefs, attachment security, interpersonal trust) with repeated assessments of contextual characteristics (e.g., family climate, organizational climate, perceived environmental safety). Longitudinal cross-lagged panel models, ecological momentary assessment, and multilevel analyses are particularly suitable for testing reciprocal influences between individuals and contexts over time.

Relevant measures may include predictive threat-related beliefs, perceived environmental safety, relational trust, attachment security, and indices of family, institutional, or community functioning. Longitudinal cross-lagged designs, structural equation modeling, ecological momentary assessment, and multilevel analyses could be used to evaluate whether changes in contextual conditions predict subsequent changes in cognitive expectations and whether these expectations, in turn, contribute to the maintenance or transformation of contextual environments. Failure to observe these reciprocal influences would challenge one of the central mechanisms proposed by CRTP.

### 3.4. Distributed Psychopathology

The third core construct introduced by the theory is distributed psychopathology.

Traditional diagnostic systems implicitly assume that psychopathology resides within individuals. Symptoms are assessed at the individual level, diagnoses are assigned to individuals, and treatment is primarily directed toward individual change. CRTP proposes an alternative conceptualization.

Distributed psychopathology refers to the emergence of psychological suffering through the interaction of multiple components distributed across persons, relationships, institutions, communities, and cultural environments.

This proposition extends ecological trauma models (Wiese, 2017), family systems approaches (Eads, 2023; Goff et al., 2020; Kelley et al., 2022), and socioecological perspectives on resilience (Ellis et al., 2012).

Under this framework, symptoms may represent indicators of systemic adaptation rather than solely personal dysfunction. Anxiety may emerge from participation in chronically unpredictable environments. Distrust may emerge from social systems organized around threat anticipation. Emotional withdrawal may emerge from relational systems in which vulnerability has historically been associated with danger. Consequently, psychopathology becomes partially contextualized. This does not deny the reality of individual suffering. Rather, it suggests that psychological symptoms often reflect broader environmental dynamics and cannot be fully understood independently of those dynamics. The individual becomes the most visible expression of a process that extends far beyond the individual.

The concept of distributed psychopathology may be illustrated across multiple social contexts. For example, elevated anxiety observed among students may not solely reflect individual vulnerability but may emerge from educational environments characterized by excessive performance pressure, chronic uncertainty, inconsistent expectations, or limited psychological safety. In such situations, anxiety becomes distributed across the student–context system rather than residing exclusively within the student.

A similar dynamic can be observed within organizations. Burnout is frequently conceptualized as an individual occupational health problem; however, many cases emerge within organizational cultures characterized by chronic overload, role ambiguity, excessive monitoring, low autonomy, or persistent uncertainty. Under these conditions, the individual’s symptoms may represent the most visible manifestation of a broader systemic adaptation to organizational demands. The pathology is therefore distributed across employees, organizational practices, leadership structures, and institutional expectations.

At the community level, historical experiences of violence, discrimination, marginalization, or collective trauma may contribute to enduring patterns of mistrust, social withdrawal, and threat anticipation. Individuals living within such environments may display elevated levels of psychological distress despite having no direct exposure to the original traumatic events. Here, vulnerability emerges through participation in a social system that continues to reproduce assumptions concerning danger, exclusion, or instability.

These examples illustrate a central proposition of CRTP: psychopathology should not always be conceptualized as a property contained within individuals. Rather, psychological symptoms may emerge from adaptive interactions occurring across multiple components of a larger system. Individuals experience the distress, but the conditions that generate and maintain that distress may be distributed throughout the contexts in which they live, learn, work, and relate to others.

Within CRTP, Distributed Psychopathology is conceptualized as a measurable property of person–context systems rather than solely of individuals. Empirical assessment may therefore combine standardized measures of individual psychological functioning (e.g., symptoms of anxiety, depression, posttraumatic stress, emotion regulation, attachment security, or interpersonal trust) with contextual indicators measured at the family, organizational, institutional, or community level, including family climate, organizational functioning, institutional safety, social cohesion, and perceived environmental unpredictability. Contextual variables may be assessed using standardized questionnaires, structured observational methods, organizational or institutional records, and community-level indicators, depending on the level of analysis. Appropriate analytical approaches include multilevel modeling, structural equation modeling, and social network analysis, allowing researchers to estimate the proportion of variance in psychological outcomes explained by contextual characteristics beyond individual-level factors alone. Conversely, if psychopathological outcomes are consistently explained exclusively by individual variables after contextual influences have been adequately modeled, the distributed psychopathology hypothesis would require revision.

An important conceptual clarification concerns the use of the term psychopathology within CRTP. The present framework does not assume that every trauma-related adaptation represents psychopathology. On the contrary, many responses to dangerous, unpredictable, or chronically stressful environments, including heightened vigilance, emotional restraint, distrust, or avoidance, are initially adaptive survival strategies that may remain functional under conditions resembling those in which they originally emerged. Within CRTP, the term psychopathology is reserved for situations in which these adaptive patterns become persistent, inflexible, and contextually mismatched, resulting in clinically significant distress, functional impairment, or substantial disruption of psychological, interpersonal, occupational, or social functioning. Likewise, vulnerability should not be equated with disorder. Individuals may carry trauma-shaped expectations or heightened sensitivity to threat without meeting diagnostic criteria for a mental disorder. The framework therefore distinguishes between adaptive contextual calibration, enduring vulnerability, and clinically significant psychopathology, viewing the latter as one possible—but not inevitable—outcome of prolonged recursive interactions between individuals and trauma-shaped environments.

### 3.5. The Contextual Transmission Cycle

**Figure 1:**
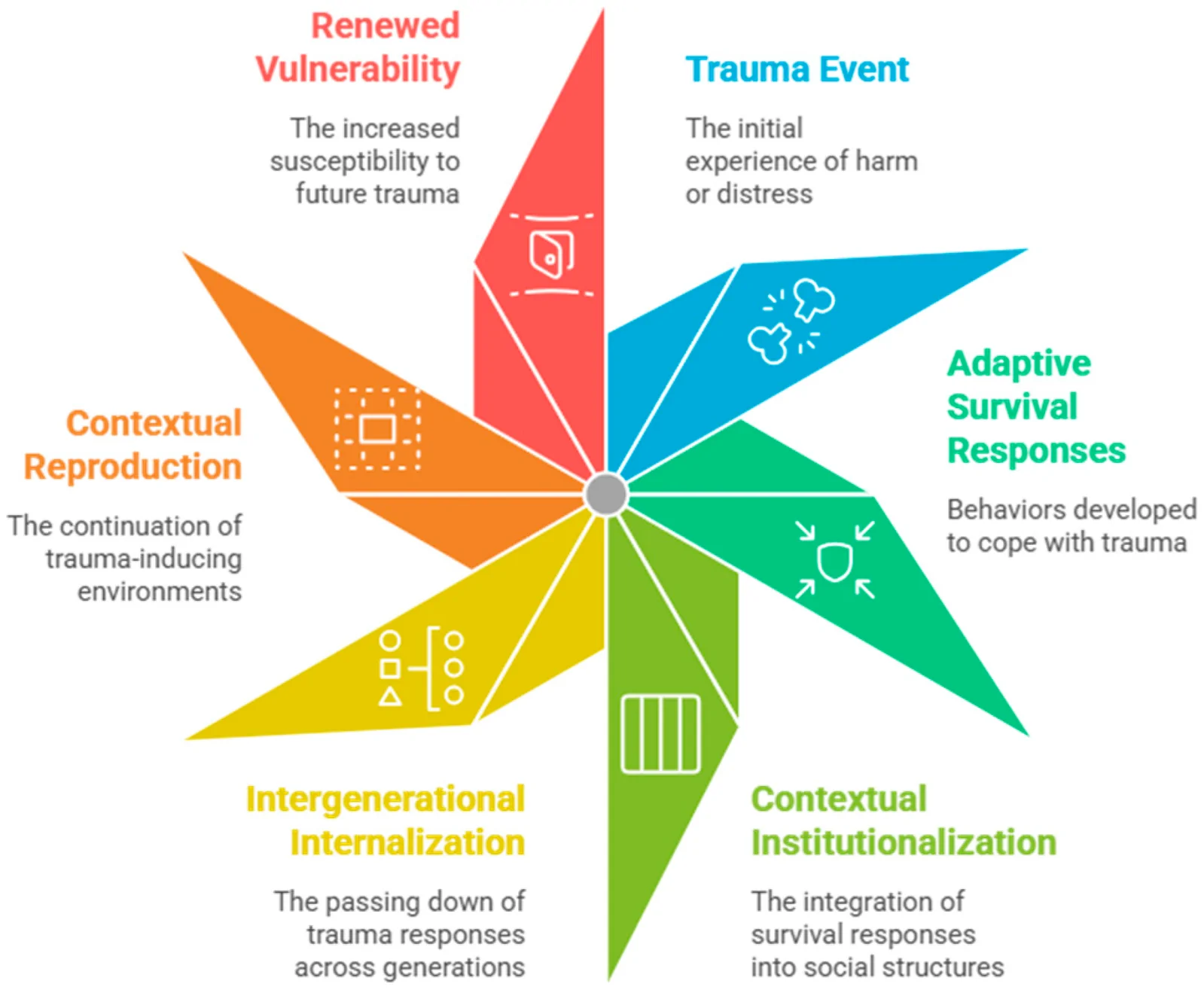
Recursive contextual transmission model.

The central mechanism of the Contextual Resonance Theory of Psychopathology is represented by the Recursive Contextual Transmission Model (Figure 1). The model proposes a six-stage cycle through which trauma becomes self-perpetuating across generations.

The cycle begins with a Trauma Event, representing exposure to adversity, violence, loss, displacement, abuse, oppression, or other forms of significant threat. In response, individuals develop Adaptive Survival Responses. These include cognitive, emotional, physiological, and behavioral adaptations designed to increase safety and predictability. Consistent with neurodevelopmental and resilience research, these responses are initially functional and adaptive (Benight, 2012; Konner, 2007; Perry & Pollard, 1998).

Over time, repeated adaptations become embedded within broader systems through a process of Contextual Institutionalization. Survival strategies are transformed into family rules, social norms, organizational practices, and cultural expectations.

Subsequently, these contextual adaptations undergo Intergenerational Internalization. New generations encounter environments already organized around assumptions established by previous experiences of adversity. Through socialization and repeated exposure, these assumptions become incorporated into individual predictive models.

The next stage involves Contextual Reproduction. Individuals who have internalized trauma-shaped assumptions contribute to recreating similar environments through their own behaviors, relationships, institutions, and cultural practices.

Finally, the cycle produces Renewed Vulnerability. Subsequent generations become increasingly susceptible to psychological distress because they continue developing within contexts organized around previous adaptations to threat.

The renewed vulnerability generated by the system increases the likelihood that future adversities will reactivate and reinforce the entire cycle, thereby initiating a new round of contextual transmission. Thus, trauma persists not because it is directly inherited, but because the environments shaped by trauma continue to exist, continue to teach, and continue to resonate across generations.

Figure 1 illustrates this recursive process and serves as the central explanatory mechanism of the Contextual Resonance Theory of Psychopathology.

To illustrate the dynamics proposed by the Contextual Resonance Theory of Psychopathology, consider the global COVID-19 pandemic as a contemporary example of how trauma may persist through contexts rather than through direct transmission of traumatic experiences. The pandemic represented a large-scale collective stressor characterized by uncertainty, fear of illness and death, social isolation, economic instability, and the sudden disruption of everyday routines. For many individuals and communities, the most psychologically significant aspect of the crisis was not the virus itself, but the prolonged experience of unpredictability and the perceived loss of control over daily life.

In response to these conditions, individuals developed a range of adaptive survival responses. Increased vigilance regarding health risks, avoidance of social contact, heightened monitoring of information, a stronger need for control, emotional withdrawal, and greater sensitivity to uncertainty were all understandable reactions within an environment perceived as threatening. At this stage, such behaviors were adaptive rather than pathological. They helped individuals navigate a context characterized by genuine danger and uncertainty.

As the crisis continued, these adaptations gradually became embedded within broader social environments through a process of contextual institutionalization. Families established new rules regarding contact with others, hygiene, and risk management. Schools implemented stricter monitoring procedures and restructured learning environments around safety concerns. Organizations normalized remote work, increased supervision through digital technologies, and emphasized predictability and risk reduction. Community life became increasingly organized around the management of potential threats. What initially emerged as individual coping strategies gradually evolved into collective patterns of functioning.

The next stage involves intergenerational internalization. Children growing up during or immediately after the pandemic may never fully experience the intensity of fear, uncertainty, and disruption that characterized the early years of the crisis. Nevertheless, they develop within environments already shaped by these adaptations. Through everyday interactions, parental behaviors, institutional practices, and social norms, they learn implicit assumptions about the world. They may come to view uncertainty as inherently dangerous, perceive social interactions as potential sources of risk, or associate safety with heightened control and constant preparedness. Importantly, these beliefs are acquired not through direct exposure to the original traumatic event but through participation in contexts that have already been reorganized around it.

As these individuals mature, they contribute to the reproduction of similar contexts. Having internalized assumptions emphasizing caution, predictability, and threat anticipation, they may become highly controlling parents, risk-averse leaders, or participants in organizations that prioritize monitoring and regulation over flexibility and trust. Without consciously intending to do so, they recreate environments that reflect the adaptive logic inherited from the previous generation. In this way, the context itself becomes the primary vehicle through which trauma-related adaptations continue to spread.

The final outcome is renewed vulnerability. The subsequent generation may exhibit elevated anxiety, reduced tolerance for uncertainty, excessive risk perception, social withdrawal, or chronic anticipation of future threats despite having no direct connection to the original crisis. When confronted with new challenges, whether economic instability, political uncertainty, technological disruption, or future public health emergencies, these individuals may be particularly susceptible because they have developed within environments that continuously reinforced expectations of danger and unpredictability.

From the perspective of the Contextual Resonance Theory of Psychopathology, the crucial observation is that the second generation does not inherit the trauma of the pandemic itself. Rather, it inherits a pandemic-shaped context. The virus may disappear, lockdowns may end, and everyday life may appear to return to normal, yet the assumptions generated by the crisis may remain embedded within families, institutions, and communities. The environment continues to communicate messages such as “the world is fundamentally unpredictable,” “control prevents catastrophe,” “trust cautiously,” and “prepare for the next threat.” Although the original traumatic event has ended, the context continues to reproduce the psychological conditions that the event initially created. In this sense, trauma persists not because it is directly transmitted from one individual to another, but because the environments shaped by trauma continue to exist, continue to teach, and continue to resonate across generations.

### 3.6. Boundary Conditions of the Contextual Resonance Theory of Psychopathology

As with any integrative theoretical framework, the Contextual Resonance Theory of Psychopathology is not intended to provide a universal explanation for all forms of psychological suffering. Rather, the theory is designed to explain a specific class of phenomena characterized by the persistence of trauma-related vulnerability beyond direct exposure to adversity and by the continued reproduction of maladaptive adaptations through environmental systems.

The theory is most applicable in situations where three conditions are simultaneously present. First, the psychological consequences of trauma persist after the original traumatic event has disappeared. Second, contextual reproduction is evident, such that family, organizational, institutional, or community environments continue to reflect adaptations originally developed in response to threat. Third, vulnerability emerges across multiple levels of analysis, including individual functioning, relational dynamics, social systems, and cultural practices.

Consequently, CRTP may be particularly useful for understanding transgenerational trauma, historical trauma, collective trauma, institutional trauma, chronic family dysfunction, and forms of psychopathology maintained through enduring environmental conditions. The framework may also help explain why vulnerability frequently persists within communities, organizations, and families despite the absence of ongoing direct exposure to the original source of adversity.

At the same time, the theory is less applicable to conditions in which contextual reproduction does not appear to play a significant role. Acute stress reactions immediately following traumatic exposure, isolated single-event trauma responses, and psychological difficulties that emerge primarily from discrete contemporary stressors may be more adequately explained by existing cognitive, neurobiological, or trauma-processing models. Similarly, CRTP is not intended to explain disorders whose primary etiological mechanisms are strongly associated with neurodevelopmental, neurological, or genetic processes independent of contextual reinforcement.

The present framework therefore should not be interpreted as a replacement for biological, attachment-based, family systems, or predictive processing accounts. Instead, it is proposed as a complementary explanatory model addressing a specific theoretical gap: the persistence and reproduction of trauma-related vulnerability through trauma-shaped contexts across time and generations. Its principal contribution lies not in explaining all psychopathology, but in explaining why certain forms of suffering continue to emerge even when the original trauma has long since disappeared.

## 4. Predictive Processing and Contextual Pathogenicity

One of the central propositions of the Contextual Resonance Theory of Psychopathology is that trauma persists across generations because it continuously shapes the predictive models through which individuals interpret reality. While the previous sections established that trauma-related adaptations may become embedded within environments, the present section seeks to explain the cognitive mechanism through which these environments exert their influence. To accomplish this, the theory draws upon the predictive processing framework, one of the most influential developments in contemporary cognitive neuroscience.

Predictive processing provides a particularly useful foundation for understanding transgenerational trauma because it shifts the focus from passive perception to active prediction. Rather than responding to reality as it unfolds, the brain continuously generates expectations about what is likely to happen next and updates these expectations based on incoming sensory information. From this perspective, psychological functioning depends fundamentally on the relationship between prediction and experience. Consequently, if trauma repeatedly alters environmental conditions, it may also alter the predictive models that individuals use to navigate the world. The Contextual Resonance Theory extends this idea by proposing that trauma-shaped environments become long-term generators of maladaptive predictions that persist across generations.

### 4.1. The Brain as a Prediction-Generating System

Predictive processing models propose that the primary function of the brain is not simply to react to sensory information but to anticipate it. Clark (2015) describes predictive processing as a canonical cortical computation in which neural systems continuously generate predictions about incoming inputs and compare those predictions against actual sensory signals. Perception therefore emerges from an ongoing dialogue between expectations and experience rather than from the passive reception of environmental information.

This perspective has been further elaborated by Benight (2012), whose formulation of Radical Predictive Processing argues that the brain functions fundamentally as a prediction machine. According to Walsh et al. (2020), perception, cognition, emotion, and action are organized around hierarchical generative models that continuously attempt to minimize discrepancies between expected and observed states of the world. The goal of the system is not objective representation but efficient prediction.

Empirical evidence supporting this framework has accumulated rapidly. Swanson (2016) reviewed neurophysiological findings and concluded that predictive processing provides a compelling account of how perception is organized across multiple levels of cortical functioning. This review suggests that predictive mechanisms are deeply embedded within neural architecture and may represent a fundamental organizing principle of cognition.

The philosophical foundations of this perspective extend even further. Williams (2018) traces predictive processing back to Kantian ideas concerning the active role of the mind in structuring experience. Rather than passively receiving reality, individuals continuously participate in constructing it through prior expectations and interpretative frameworks. Similarly, Wiese (2017) and Wilkinson et al. (2017) emphasize that predictive processing challenges traditional representational views of cognition by suggesting that mental representations are fundamentally anticipatory, organized around expectations regarding future states rather than static descriptions of present reality.

These perspectives converge on a central principle: human beings do not simply perceive the world they inhabit. They perceive the world they expect to inhabit. This observation is particularly important for understanding trauma. If perception depends on prediction, then traumatic experiences may alter not only how individuals remember the past but also how they anticipate the future.

### 4.2. Trauma as Predictive Calibration

Within predictive processing frameworks, trauma can be understood as a process of predictive calibration. Rather than conceptualizing trauma exclusively as emotional injury or psychological damage, several authors have proposed that traumatic experiences fundamentally modify the brain’s predictive models concerning safety, threat, trust, and control.

Krupnik (2020) argues that psychological trauma may be understood as a disruption of predictive systems responsible for modeling environmental regularities. Exposure to overwhelming threat generates powerful learning experiences that recalibrate expectations regarding future events. The individual learns that danger is more probable, safety is less predictable, and uncertainty carries greater potential costs.

Putica and Agathos (2024) similarly propose that trauma exists along a continuum of predictive adaptation. From this perspective, stress responses emerge when environmental demands exceed the predictive capacities of existing models. Severe trauma produces enduring recalibrations that prioritize threat detection and defensive responding. Importantly, these changes are not irrational; they represent adaptive attempts to minimize future surprise under conditions where danger has become highly salient.

Contemporary trauma models increasingly reflect this predictive perspective. Sopp et al. (2022) argue that Complex PTSD can be reconceptualized as a disorder of maladaptive predictive models. Repeated exposure to interpersonal trauma generates persistent expectations concerning danger, rejection, helplessness, and loss of control. These expectations subsequently shape perception, emotional regulation, interpersonal functioning, and behavioral decision-making.

Research examining trauma and belief updating further supports this interpretation. Haim-Nachum et al. (2024) found that trauma exposure influences how individuals revise expectations in response to new information. Trauma survivors often show difficulties updating previously learned threat-related beliefs even when environmental conditions change. Similarly, Griffin and Fletcher (2017) demonstrated that trauma-exposed individuals may struggle to incorporate disconfirmatory evidence regarding safety, suggesting that threat expectations become resistant to modification.

These findings suggest that trauma does not simply produce symptoms. It recalibrates the predictive architecture through which reality itself is interpreted.

### 4.3. Context as a Generator of Chronic Prediction Error

While existing predictive models have primarily focused on how traumatic events influence individual predictive systems, the Contextual Resonance Theory introduces an additional proposition: environments themselves may become generators of maladaptive predictive calibration.

Within predictive processing frameworks, prediction errors arise whenever incoming information differs from expectations (Clark, 2015; Walsh et al., 2020). Under normal circumstances, prediction errors facilitate learning by encouraging individuals to update inaccurate beliefs. However, environments characterized by chronic instability, unpredictability, inconsistency, or threat may repeatedly generate prediction errors that reinforce defensive expectations rather than correcting them.

Consider a child growing up within a family shaped by unresolved trauma. Emotional availability may fluctuate unpredictably. Rules may change without explanation. Expressions of vulnerability may sometimes be met with support and other times with rejection. Trust may be inconsistently rewarded. Under such conditions, the child is exposed not merely to isolated stressful experiences but to a context that continuously undermines the formation of stable predictive models.

Over time, uncertainty itself becomes predictable. The individual learns not that the world is dangerous in a specific way, but that safety can never be fully anticipated.

This distinction is critical. Trauma-shaped environments may function as chronic generators of ambiguity, producing continuous discrepancies between expectations and outcomes. As a consequence, individuals increasingly rely upon defensive predictions organized around vigilance, caution, control, and threat anticipation.

Importantly, these contextual influences may persist independently of direct trauma exposure. Even when the original traumatic event has disappeared, environments shaped by previous adaptations continue generating experiences that reinforce particular predictive expectations. Thus, psychopathology becomes maintained not only by internal cognitive processes but also by the environments that repeatedly validate those processes.

### 4.4. Contextual Resonance and Threat Expectation

The concept of contextual resonance represents the central predictive mechanism of the Contextual Resonance Theory of Psychopathology. Contextual resonance occurs when environmental conditions repeatedly confirm trauma-related predictions, thereby increasing the stability and persistence of maladaptive predictive models. Rather than correcting inaccurate expectations, the environment continuously provides experiences that appear to validate them.

This process creates a self-reinforcing feedback loop. Trauma-related expectations influence perception and behavior. These behaviors contribute to the maintenance of trauma-shaped environments. Those environments subsequently generate experiences that confirm existing expectations. The cycle then repeats.

The concept bears similarities to predictive accounts of psychosis proposed by Kotler et al. (2026), who describe psychopathology as emerging from disturbances in predictive inference and source monitoring processes. However, the present framework extends this logic beyond individual cognition by emphasizing the role of contexts in stabilizing particular forms of predictive processing.

Recent developments within trauma theory further support this extension. Fujimoto (2026) argues that trauma should be understood not as a static memory stored within the body but as a disruption of adaptive predictive dynamics. Recovery involves restoring flexibility, adaptability, and metastability within predictive systems. Similarly, Bronfenbrenner (1994) proposes that body-oriented trauma interventions may facilitate recovery by creating novel sensory experiences capable of updating deeply entrenched predictive models.

From the perspective of CRTP, these interventions succeed precisely because they interrupt contextual resonance. They expose individuals to experiences that systematically violate trauma-related expectations and provide opportunities for predictive recalibration.

The crucial implication is that trauma-related vulnerability may persist not because traumatic memories remain active, but because environments continue to reinforce trauma-consistent predictions. Families, communities, organizations, and institutions can therefore become powerful amplifiers of threat expectation, repeatedly communicating messages concerning danger, distrust, unpredictability, exclusion, or lack of control.

Consequently, transgenerational trauma may be understood as the intergenerational transmission of predictive environments. Individuals inherit contexts that teach them what to expect from the world. These expectations subsequently shape perception, emotion, behavior, and future contextual reproduction. In this sense, contextual resonance represents the cognitive engine through which trauma-shaped environments continue to exert influence across generations. Trauma survives not only because it is remembered, but because the environments shaped by trauma repeatedly teach individuals to anticipate its return.

## 5. Corrective Recontextualization: A Proposed Mechanism of Recovery

If the Contextual Resonance Theory of Psychopathology is correct in proposing that trauma persists through the recursive reproduction of trauma-shaped environments, then recovery cannot be conceptualized exclusively as an intrapsychic process. Traditional trauma interventions have understandably focused on reducing symptoms, processing traumatic memories, modifying maladaptive beliefs, or improving emotional regulation. While such approaches remain essential, the present framework suggests that they address only part of the problem. If psychological vulnerability is continuously reinforced by contextual conditions, then lasting recovery requires not only changes within the individual but also changes in the environments through which experience is interpreted, predicted, and enacted.

An important implication of this perspective is that trauma-shaped contexts are inherently paradoxical rather than uniformly pathogenic. The same institutional domains capable of sustaining trauma, including families, schools, religious organizations, healthcare systems, workplaces, and broader communities, may also become powerful sources of psychological recovery when reorganized around safety, predictability, trust, and supportive social relationships. Whether a given context amplifies or attenuates trauma therefore depends less on its formal structure than on the quality of the recursive interactions, meanings, and adaptive expectations that it continuously reproduces. From the perspective of CRTP, psychopathology and recovery should not be understood as properties of particular institutions themselves, but as emergent outcomes of the contextual organization operating within those institutions. Consequently, corrective recontextualization does not require replacing one social context with another; rather, it involves the gradual transformation of the relational, cognitive, and institutional dynamics through which contexts become either trauma-maintaining or psychologically restorative (J. C. Alexander, 2012; Kaiser & Butler, 2015; Rothbaum et al., 2002).

The process of corrective recontextualization also implies an important shift in human agency. Rather than remaining constrained by adaptive patterns established in response to past traumatic environments, individuals gradually develop the capacity to construct alternative trajectories through repeated participation in psychologically restorative contexts. In this sense, corrective recontextualization reflects a transition from predominantly iterational forms of agency, in which present action is guided by previously established trauma-related expectations, toward more projective forms of agency oriented to future possibilities and adaptive reorganization (Emirbayer & Mische, 1998). CRTP therefore conceptualizes recovery not simply as symptom reduction but as the progressive expansion of an individual’s capacity to participate in and actively co-construct contexts that support safety, trust, flexibility, and psychological growth. Agency is thus understood as emerging within recursive interactions between individuals and their environments rather than as an exclusively internal personal attribute.

To address this challenge, the theory proposes corrective recontextualization as a complementary mechanism of recovery. Corrective recontextualization refers to the sustained exposure to environments that systematically challenge trauma-shaped expectations and provide opportunities for the construction of alternative predictive models concerning safety, trust, belonging, autonomy, and relational stability. Recovery, in this view, emerges not solely through remembering the past differently, but through repeatedly experiencing the present differently.

Recent trauma-responsive peacebuilding approaches likewise emphasize that sustainable recovery requires interventions targeting the environments in which individuals live rather than focusing exclusively on intrapsychic change. Trauma-aware educational, community, and institutional practices have increasingly been recognized as essential for reducing the long-term reproduction of trauma across generations (Reimann & Clarke-Habibi, 2026). These developments closely align with the principle of Corrective Recontextualization proposed within CRTP.

Within CRTP, Corrective Recontextualization is conceptualized as a recursive adaptive process rather than a discrete therapeutic technique. Trauma-shaped predictive models are maintained because individuals repeatedly encounter contexts that confirm previously acquired expectations regarding danger, rejection, unpredictability, or helplessness. Corrective contexts interrupt this cycle by providing repeated experiences that consistently violate these maladaptive predictions while simultaneously offering coherent and emotionally meaningful alternatives. Through the accumulation of prediction errors across multiple interactions, existing cognitive models are progressively revised, allowing new expectations about the self, others, and the environment to emerge and stabilize.

Importantly, Corrective Recontextualization extends beyond the notion of a supportive environment. While emotional support, empathy, and interpersonal safety are necessary components of recovery, they are not sufficient to modify deeply established predictive structures if they remain isolated experiences. A corrective context is characterized by the systematic organization of relational, educational, institutional, occupational, and community environments that repeatedly reinforce adaptive expectations through consistent experiences across multiple ecological levels. Consequently, recovery is promoted not by isolated positive interactions but by the sustained coherence of contextual experiences that collectively facilitate cognitive reorganization.

Within this perspective, durable psychological change is expected to result from the progressive stabilization of newly acquired predictive models through repeated contextual confirmation. As adaptive expectations are continuously reinforced across different domains of everyday life, they gradually become more resistant than previously established trauma-related predictions. Recovery is therefore understood not as the elimination of traumatic memories but as the development of increasingly stable contextual conditions that support adaptive prediction, emotional regulation, interpersonal functioning, and psychological flexibility over time.

Accordingly, Corrective Recontextualization represents the principal translational mechanism of the Contextual Resonance Theory of Psychopathology. Rather than focusing exclusively on symptom reduction, CRTP proposes that sustainable recovery requires the intentional reorganization of the contexts within which individuals think, relate, learn, work, and participate in society. By integrating therapeutic interventions with supportive relational, institutional, and sociocultural environments, the theory provides a multilevel framework through which maladaptive contextual resonance may gradually be transformed into adaptive contextual functioning across generations.

Several important questions regarding Corrective Recontextualization remain to be addressed empirically, including the optimal duration and intensity of corrective contextual exposure, individual characteristics that may moderate its effectiveness, and the potential unintended consequences of unsuccessful or inconsistent corrective environments. These questions represent important priorities for future research.

### 5.1. Moving Beyond Symptom Reduction

For much of the history of trauma treatment, therapeutic success has been evaluated primarily in terms of symptom reduction. Improvements in anxiety, hyperarousal, avoidance, intrusive memories, or emotional distress have often been treated as indicators of recovery. Although these outcomes remain clinically important, a growing body of literature suggests that recovery from trauma involves broader adaptive processes that cannot be fully captured through symptom-focused frameworks alone.

Adaptation to traumatic stress should be understood as a process of restoring perceived efficacy and adaptive functioning rather than simply eliminating symptoms (Konner, 2007). From this perspective, recovery involves rebuilding the individual’s capacity to navigate uncertainty, engage with challenges, and maintain meaningful participation in life. Research on resilience further supports this shift in emphasis. Successful adaptation depends not merely on individual coping capacities but on access to environments that provide opportunities for safety, support, belonging, and growth (Ellis et al., 2012). Consequently, reducing symptoms without altering the contextual conditions that sustain vulnerability may leave important mechanisms of distress intact.

The present theory extends these observations by suggesting that recovery should be evaluated not only in terms of what symptoms disappear, but also in terms of what predictive possibilities become available. The critical question is not simply whether individuals feel less distressed, but whether they are able to construct fundamentally different expectations about themselves, others, and the world.

### 5.2. Relearning Safety Through Environmental Exposure

One of the central implications of predictive processing is that expectations are not modified primarily through insight alone. They are modified through experience. Individuals learn safety in much the same way they learn danger: through repeated encounters with environmental regularities that become incorporated into predictive models.

Trauma often produces persistent expectations that the world is unpredictable, that vulnerability leads to harm, that trust invites betrayal, or that safety is temporary and fragile. These expectations may remain remarkably stable because trauma-shaped environments continue to provide experiences that appear to confirm them. Consequently, recovery requires more than challenging beliefs cognitively; it requires exposure to contexts capable of generating alternative experiences.

This proposition finds support within contemporary trauma intervention research. Improvements in trauma-related functioning were strongly associated with changes in environmental conditions, including greater relational stability, increased support, and improved contextual safety (Lange et al., 2022). These findings suggest that therapeutic progress is facilitated when environments themselves become part of the intervention process.

Similarly, Raja and Carrico (2022) observed that effective trauma-focused interventions frequently require adaptation to the individual’s social, developmental, familial, and cultural circumstances. Such findings highlight a broader principle: psychological healing is often inseparable from the contexts within which it occurs.

Corrective recontextualization therefore involves repeated participation in environments that violate trauma-based expectations. Consistent emotional availability challenges expectations of abandonment. Predictable social interactions challenge expectations of unpredictability. Supportive relationships challenge expectations of rejection. Institutional fairness challenges expectations of exploitation. Over time, these experiences provide the informational basis necessary for updating deeply entrenched predictive models.

Importantly, the process is gradual. Trauma-related expectations are often the product of years of accumulated learning. Consequently, safety must become sufficiently frequent, consistent, and predictable to acquire comparable predictive value.

### 5.3. Recalibration of Predictive Models

Within predictive processing frameworks, recovery may be conceptualized as a process of predictive recalibration. Trauma alters expectations concerning danger, uncertainty, and control. Healing requires the development of alternative models that better reflect current environmental realities.

Recent predictive processing approaches to trauma strongly support this interpretation. Trauma may be understood as a disturbance in predictive systems governing perception and meaning-making (Krupnik, 2020). Similarly, Sopp et al. (2022) propose that many features of Complex PTSD reflect rigid predictive models that continuously anticipate threat, rejection, and loss of control even in objectively safe environments.

Research examining belief updating provides important insight into this process. Trauma-related difficulties are associated with reduced flexibility in updating expectations following new experiences (Haim-Nachum et al., 2024). Likewise, Griffin and Fletcher (2017) found that trauma-exposed individuals often struggle to incorporate evidence that contradicts existing danger-related beliefs. Safety information may be discounted, ignored, or interpreted through pre-existing threat expectations.

From the perspective of corrective recontextualization, recovery occurs when environments provide sufficient opportunities for successful predictive updating. New experiences gradually acquire enough weight to challenge older assumptions. The individual begins to encounter evidence that trust is sometimes rewarded, vulnerability is sometimes safe, uncertainty is sometimes manageable, and relationships are not inevitably dangerous.

This process does not erase previous learning. Rather, it expands the repertoire of available predictions. Threat remains possible, but it is no longer the only expected outcome.

Recent theoretical developments further reinforce this perspective. Fujimoto (2026) conceptualizes trauma recovery as the restoration of metastability, namely, the capacity of predictive systems to flexibly adapt to changing environmental demands. Similarly, Bronfenbrenner (1994) argues that trauma interventions may be effective precisely because they create novel experiential conditions capable of disrupting rigid predictive patterns and facilitating the emergence of alternative modes of engagement with the world.

### 5.4. Breaking Transgenerational Transmission Pathways

The final implication of corrective recontextualization concerns transgenerational transmission itself. If trauma persists through the recursive reproduction of trauma-shaped environments, then interrupting transmission requires altering the contexts through which future generations learn to interpret reality.

Traditional approaches to transgenerational trauma have often focused on processing historical experiences, increasing awareness of family narratives, or addressing individual symptoms among descendants. While these strategies remain valuable, the present theory suggests that the most powerful intervention may occur at the level of context.

A parent who develops greater emotional availability creates a different developmental environment for a child. A family that replaces chronic vigilance with predictability alters the learning conditions through which future expectations are formed. Schools that promote psychological safety, communities that foster social trust, and institutions that provide consistency and fairness may all function as corrective environments capable of weakening the contextual reproduction of trauma-related adaptations.

This perspective aligns closely with ecological understandings of resilience. Resilience emerges when environments provide resources that support healthy adaptation (Ellis et al., 2012). Extending this principle, corrective recontextualization proposes that resilience may become transmissible when supportive contexts become stable features of family, community, and institutional life.

The ultimate goal is not merely the reduction of vulnerability within a single generation. Rather, it is the creation of contexts that no longer require trauma-based adaptations. When environments consistently communicate safety, predictability, trustworthiness, and belonging, the recursive cycle described in the Contextual Resonance Theory begins to weaken. Contextual traumatic inertia diminishes, contextual resonance shifts toward adaptive expectations, and the reproduction of trauma-shaped environments becomes less likely.

From this perspective, healing represents more than recovery from past adversity. It represents the gradual transformation of the environments through which future generations learn what to expect from the world. In this way, corrective recontextualization offers a potential mechanism through which the intergenerational persistence of trauma may ultimately be interrupted, not by changing the past, but by changing the contexts that continue to give the past predictive power in the present.

**Figure 2:**
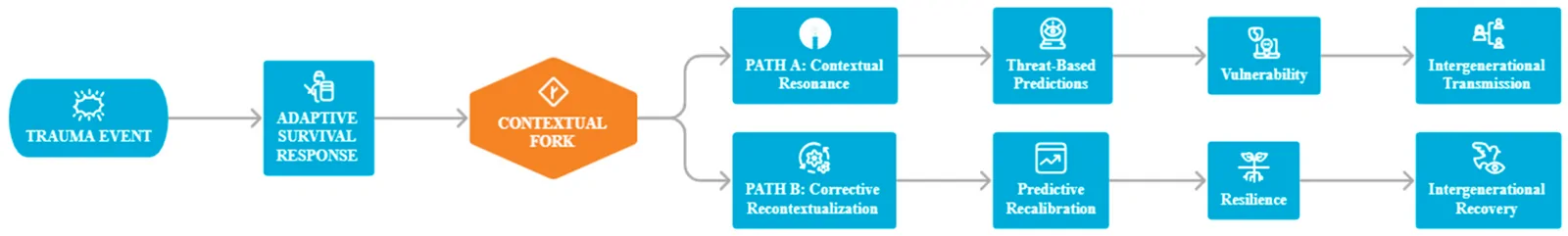
Dual pathway model of contextual resonance and corrective recontextualization.

The recursive transmission cycle described in Figure 1 emphasizes the mechanisms through which trauma-shaped adaptations persist across generations. However, the same framework also implies the possibility of developmental divergence. Exposure to trauma-shaped environments is not the only possible outcome following adversity. Under certain conditions, adaptive responses may be followed by corrective environmental experiences capable of interrupting contextual reproduction and facilitating resilience. Figure 2 presents a dual-pathway model illustrating how contextual resonance and corrective recontextualization may lead to contrasting developmental trajectories.

Following a traumatic event, adaptive survival responses may evolve along two alternative contextual trajectories. Trauma-shaped environments promote contextual resonance and the persistence of threat-based predictive models, increasing vulnerability across generations. In contrast, corrective environments facilitate predictive recalibration, resilience, and the interruption of transgenerational transmission pathways.

## 6. Discussion

The present paper was motivated by a persistent conceptual challenge within the literature on transgenerational trauma. Despite substantial advances across biological, psychological, relational, and sociocultural domains, no existing framework fully explains why trauma-related vulnerability frequently persists long after the original traumatic event has disappeared. Genetic and epigenetic models have identified potential biological pathways of transmission, attachment-based approaches have highlighted the role of relational experiences and internal working models, while family systems and socioecological perspectives have emphasized multigenerational relational processes and contextual influences (Eads, 2023; Kelley et al., 2022). Yet a central question remains unresolved: what exactly continues to transmit vulnerability when biological inheritance is incomplete, explicit memories have faded, and the original traumatic circumstances no longer exist?

Importantly, the present framework is not intended as a general theory of psychopathology but as a theory of context-mediated persistence of trauma-related vulnerability across generations and social systems. Distributed psychopathology does not imply that suffering is unreal or that responsibility is removed from the individual. Rather, it proposes that the determinants of suffering may be distributed across multiple interacting components of a social system, making exclusively individual-level explanations incomplete. Another important aspect is that although contexts strongly constrain adaptation, CRTP does not assume deterministic effects. Individuals remain active agents capable of modifying, resisting, and transforming contextual influences, thereby creating opportunities for corrective recontextualization and resilience.

The Contextual Resonance Theory of Psychopathology was developed as a response to this question. Rather than conceptualizing trauma as something transmitted directly between individuals, the theory proposes that trauma persists through the recursive reproduction of contexts organized around adaptations to previous adversity. In doing so, the theory does not seek to replace existing explanations but to integrate and extend them by introducing context itself as the primary unit of transmission.

This position differs in important ways from contemporary epigenetic models. Research by Chou et al. (2024), Ghai and Kader (2022), Gill et al. (2026), Jawaid et al. (2018), and Yehuda et al. (2018) has substantially advanced understanding of how traumatic experiences may influence biological systems across generations. Similarly, Chou et al. (2024), Gill et al. (2026), Liotti (2006), Salberg (2016), and Yehuda et al. (2018) have provided valuable insights into the complex interactions between environmental stressors and gene expression. However, even the strongest epigenetic accounts acknowledge that biological mechanisms alone cannot explain the full variability observed in trauma outcomes. Individuals sharing similar biological vulnerabilities frequently display markedly different developmental trajectories depending on the environments in which they live. The present framework therefore suggests that biological adaptations may contribute to vulnerability, but their long-term significance depends largely on the contexts that either reinforce or modify them. In this sense, contextual resonance may function as a complementary mechanism that helps explain why some trauma-related adaptations persist while others gradually diminish.

The proposed framework also extends attachment-based explanations of transgenerational trauma. Attachment theory has consistently demonstrated that early relational experiences shape expectations regarding safety, trust, emotional regulation, and interpersonal functioning (Abrams, 1999; Farina & Schimmenti, 2025; Kostova & Matanova, 2024; Meulewaeter et al., 2019). The Contextual Resonance Theory fully acknowledges these contributions but argues that attachment relationships themselves are embedded within broader contextual systems. Parents do not transmit attachment patterns in isolation; they do so within environments already organized by historical experiences, cultural norms, institutional structures, and family narratives. Consequently, attachment insecurity may be understood not only as a dyadic phenomenon but also as a contextual one. Trauma-shaped environments influence caregivers, caregivers influence children, and children subsequently contribute to the reproduction of those environments, creating recursive cycles that extend beyond individual relationships.

Similarly, the theory builds upon ecological and family systems approaches while introducing a more explicit explanatory mechanism for persistence across generations. Research by Ellis et al. (2012), Lange et al. (2022), Raja and Carrico (2022), and Ungar (2013) emphasizes the importance of environmental influences on trauma and adaptation. However, these frameworks primarily describe the existence of contextual effects rather than explaining how contexts themselves become carriers of traumatic adaptation. The concept of contextual traumatic inertia was developed precisely to address this issue. It proposes that environments possess a tendency to preserve and reproduce adaptations that were once functional under conditions of threat. This process may help explain why families, organizations, institutions, and communities often continue operating according to assumptions established during previous periods of adversity even when those adversities are no longer present.

An important distinction should be drawn between the present framework and the ecological systems approaches described by J. C. Alexander (2012), Emirbayer and Mische (1998), Kaiser and Butler (2015), and Rothbaum et al. (2002). At first glance, both perspectives emphasize the importance of environmental influences on human development, and both reject exclusively individualistic explanations of psychological functioning. However, the two frameworks address fundamentally different questions. Ecological Systems Theory primarily explains how nested environmental systems influence developmental outcomes. The central assumption is that development emerges through interactions between individuals and multiple contextual layers, ranging from immediate family relationships to broader sociocultural structures. From this perspective, the framework described by Rothbaum et al. (2002) offers a comprehensive explanation of contextual influences on development. The Contextual Resonance Theory of Psychopathology, by contrast, seeks to explain contextual persistence. Rather than asking how environments influence development, CRTP asks how environments themselves become shaped by trauma and continue reproducing trauma-related adaptations across time. The primary focus is therefore not on the existence of contextual effects, but on the mechanisms through which contexts acquire, preserve, and transmit adaptive responses originally developed under conditions of adversity. A second distinction concerns the role of historical continuity. Within Ecological Systems Theory, environments influence development, but the environments themselves are not conceptualized as carriers of inherited adaptations. In CRTP, contexts possess a form of functional memory. Family systems, organizations, communities, and institutions may continue operating according to assumptions established during previous periods of threat long after the original traumatic conditions have disappeared. This process is captured by the concept of contextual traumatic inertia. Finally, although both theories acknowledge bidirectional interactions between individuals and environments, CRTP introduces the concept of recursive contextual resonance to explain how trauma-related expectations become continuously reinforced through feedback loops linking persons and contexts. Individuals shape environments, environments shape individuals, and the resulting adaptations contribute to the reproduction of the same contextual conditions that generated them. Thus, while Ecological Systems Theory explains why context matters, CRTP attempts to explain how trauma-shaped contexts persist, reproduce themselves, and continue influencing future generations.

A similar distinction should be drawn between the Contextual Resonance Theory of Psychopathology and Family Systems Theory. Family systems approaches have made indispensable contributions to understanding transgenerational trauma by demonstrating that traumatic experiences become embedded within relational structures, communication patterns, emotional roles, and multigenerational family processes (Bachem et al., 2018; Fitzgerald et al., 2020; Goff et al., 2020; Papero, 2017). These perspectives shifted attention away from the isolated individual and toward the family as a dynamic emotional system through which vulnerability and adaptation may be transmitted across generations. The present framework fully acknowledges these contributions and builds directly upon them. However, CRTP proposes that the family represents only one possible manifestation of a broader contextual process. Trauma-shaped adaptations are not confined to family systems but may become embedded within schools, organizations, institutions, communities, and cultural environments. Consequently, the primary unit of transmission is not the family itself but the context that carries and reproduces adaptations originally developed in response to adversity. This distinction becomes particularly important when examining situations in which trauma-related patterns persist despite substantial changes in family structure or caregiving relationships. Individuals frequently encounter trauma-shaped assumptions regarding trust, authority, vulnerability, control, belonging, or threat through educational systems, workplaces, professional cultures, social institutions, and community environments. Such contexts may continue reinforcing trauma-related expectations even when family-level transmission has been interrupted. Accordingly, Family Systems Theory explains how trauma becomes organized within family relationships, whereas CRTP seeks to explain how trauma-related adaptations become distributed across multiple social systems and continue reproducing vulnerability beyond the family context. The theory therefore extends the systemic perspective from the family as the primary carrier of transmission to a broader ecology of trauma-shaped contexts operating across multiple levels of social organization.

A second novel contribution concerns the integration of predictive processing principles into the study of transgenerational trauma. Contemporary predictive models suggest that the brain continuously generates expectations about future states of the world and updates these expectations based on incoming information (Clark, 2015; Swanson, 2016; Walsh et al., 2020). Recent trauma research has increasingly applied this perspective to understanding PTSD and Complex PTSD (Krupnik, 2020; Putica & Agathos, 2024; Sopp et al., 2022). The Contextual Resonance Theory extends these developments by proposing that environments themselves function as long-term regulators of predictive learning. Contexts repeatedly communicate assumptions about safety, trust, danger, belonging, and uncertainty. Consequently, trauma transmission may be understood not merely as the inheritance of vulnerability but as the inheritance of predictive environments that shape how reality is anticipated and interpreted.

Although the primary focus of this article has been theoretical, the framework carries implications for multiple domains of intervention and future research. Within psychotherapy, the theory suggests that symptom reduction alone may be insufficient when trauma-related expectations continue to be reinforced by the individual’s environment. This observation is consistent with intervention studies demonstrating the importance of contextual change alongside individual treatment (Lange et al., 2022; Raja & Carrico, 2022). Recovery may therefore depend not only on modifying beliefs or processing memories but also on creating opportunities for sustained engagement with environments capable of generating alternative predictive experiences.

The framework also highlights the importance of family-level interventions. If trauma transmission occurs through contextual reproduction, then efforts to increase emotional availability, relational predictability, and psychological safety may have effects that extend beyond individual family members. Similar principles apply to educational settings. Schools represent powerful developmental contexts in which expectations concerning competence, trust, belonging, and social safety are continuously constructed. From the perspective of corrective recontextualization, educational environments may function either as amplifiers of trauma-shaped expectations or as contexts that facilitate their modification.

The same logic extends to organizations and communities. Institutions often inherit historical adaptations in much the same way families do. Organizations formed under conditions of chronic uncertainty may normalize excessive monitoring, rigid control, or defensive decision-making. Communities exposed to historical adversity may develop collective assumptions concerning trust, vulnerability, or exclusion that persist long after the original conditions have changed. Understanding these dynamics may contribute to the development of interventions that target contextual processes rather than focusing exclusively on individual functioning.

A further consideration concerns critical perspectives on trauma theory. Contemporary scholarship has cautioned that trauma frameworks may inadvertently medicalize adaptive responses to adversity or underemphasize the structural, historical, and sociocultural determinants of psychological suffering. Likewise, critical cultural psychology emphasizes that concepts such as distress, impairment, and adaptation are partly shaped by cultural norms and therefore cannot be assumed to possess identical meanings across societies. CRTP is intended to be consistent with these perspectives by conceptualizing trauma-related responses as initially adaptive rather than inherently pathological and by emphasizing that clinically significant psychopathology emerges only when trauma-shaped adaptations become persistent, contextually mismatched, and associated with substantial distress or functional impairment. Furthermore, the framework explicitly recognizes that contextual organization is historically, culturally, and institutionally situated, implying that both vulnerability and recovery must be interpreted within their specific sociocultural environments rather than as universally invariant psychological processes.

Several directions for future research emerge from the present framework. First, the constructs of contextual resonance and contextual traumatic inertia require empirical operationalization. Developing valid measures capable of capturing trauma-shaped environmental characteristics represents an essential next step. Second, longitudinal and multilevel research designs will be necessary to examine how contextual adaptations evolve over time and how they influence developmental trajectories across generations. Third, advances in predictive neuroscience may offer opportunities to identify neurocognitive markers associated with contextual learning, threat expectation, and predictive recalibration following exposure to corrective environments. Future research should compare the predictions of CRTP with those of alternative explanatory models, including epigenetic, historical trauma, and cultural trauma frameworks. Such comparative studies would help identify the unique explanatory contribution of contextual resonance and establish the empirical boundaries of the proposed theory. Future research should also examine the application of CRTP in migrant, refugee, and displaced populations, particularly by investigating how contextual discontinuity, cultural distance, and social integration influence processes of contextual recalibration following relocation.

Emerging technological approaches may also prove valuable. Ecological monitoring methods, digital phenotyping, and artificial intelligence systems increasingly allow researchers to analyze complex environmental patterns across multiple levels of human functioning. Such approaches may eventually provide tools for identifying trauma-shaped contexts, monitoring contextual change, and evaluating the effectiveness of corrective recontextualization interventions. Although these possibilities remain largely speculative, they represent promising directions for extending the empirical study of context beyond traditional self-report methodologies.

For a theoretical framework to contribute to cumulative scientific knowledge, its central propositions must be empirically examinable and potentially falsifiable. The Contextual Resonance Theory of Psychopathology generates a series of testable predictions concerning the persistence, transmission, and modification of trauma-related adaptations across contexts. First, the construct of contextual traumatic inertia predicts that trauma-shaped contextual characteristics should remain observable even after the original traumatic conditions have disappeared. Families, organizations, schools, or communities previously exposed to significant adversity should display greater stability of threat-related norms, expectations, and practices than comparable systems without such histories. Second, the construct of recursive contextual resonance predicts that individual threat expectations should correlate with the degree to which environmental contexts communicate cues of unpredictability, distrust, exclusion, or danger. Individuals repeatedly exposed to trauma-shaped contexts should demonstrate stronger threat-related predictive biases than individuals exposed to corrective environments. Third, the concept of corrective recontextualization predicts that sustained exposure to psychologically restorative environments should be associated with progressive reductions in maladaptive threat expectations, increased predictive flexibility, and improvements in psychosocial functioning. Such changes should occur even in the absence of direct trauma-focused interventions. Finally, the concept of distributed psychopathology predicts that contextual variables will explain unique variance in psychological outcomes beyond individual-level predictors alone. Models incorporating contextual measures should demonstrate greater explanatory power than models relying exclusively on intrapsychic or demographic variables. Depiction is presented below, in Table 3.

The hypotheses generated by the Contextual Resonance Theory of Psychopathology are intended to be empirically testable and potentially falsifiable. Accordingly, each principal prediction can be operationalized through clearly defined variables, comparison conditions, and expected empirical outcomes. Rather than assuming universal applicability, CRTP specifies conditions under which its principal propositions would be supported or challenged. Table 4 summarizes the proposed operational definitions, comparison strategies, expected findings, and potential falsification criteria for the theory’s major hypotheses.

Several limitations of the present theory should also be acknowledged. As a conceptual framework, the Contextual Resonance Theory currently lacks direct empirical validation. Many of its propositions remain hypothetical and require systematic testing. Furthermore, the theory intentionally emphasizes contextual mechanisms, creating the risk of underestimating the importance of biological, genetic, and individual differences. The intention is not to replace these factors but to complement them within a broader explanatory framework. Future research will need to clarify how contextual processes interact with biological vulnerability, personality, attachment history, and developmental timing. In addition, the boundaries of what constitutes a trauma-shaped context remain open to interpretation and will require careful conceptual refinement.

Despite these limitations, the proposed framework offers a potentially useful shift in perspective. Rather than asking how trauma moves from one individual to another, it encourages researchers to examine how environments become organized around traumatic adaptations and how these environments continue to shape expectations, perceptions, and behavior across generations. Such a shift may help bridge currently fragmented literatures spanning trauma studies, attachment theory, predictive processing, resilience research, family systems theory, and ecological psychology. More importantly, it highlights a possibility that has received relatively little attention within existing models: trauma may persist not because people continue to remember it, but because the contexts shaped by trauma continue to teach it.

Beyond its conceptual contribution, the Contextual Resonance Theory of Psychopathology also generates empirically testable predictions that distinguish it from existing theoretical models. First, CRTP predicts that trauma-related psychopathology will be more accurately explained by multilevel interactions among biological vulnerability, predictive cognitive processes, relational dynamics, and contextual characteristics than by any single explanatory mechanism considered in isolation. Second, the theory predicts that the persistence of psychopathological symptoms will depend not only on the severity of the original traumatic exposure but also on the extent to which individuals continue to be embedded in contexts that recursively reinforce trauma-related predictive models. Conversely, sustained exposure to corrective contextual conditions should predict progressive cognitive reorganization and improved psychological adaptation over time.

From a clinical perspective, CRTP suggests that assessment should extend beyond individual symptoms to include the relational, institutional, and sociocultural contexts in which those symptoms are maintained. Intervention planning may therefore target multiple levels simultaneously, combining individual psychotherapy with family, school, organizational, or community-based strategies when trauma-shaped contextual processes contribute to psychological vulnerability. The specific operationalization of these multilevel interventions represents an important direction for future clinical research.

These propositions provide multiple opportunities for empirical evaluation. Future studies may operationalize contextual resonance through multilevel indicators encompassing individual cognitive schemas, attachment quality, family functioning, institutional climate, social support, and broader sociocultural conditions. Longitudinal and multilevel research designs would be particularly appropriate for examining the dynamic interactions proposed by CRTP, while structural equation modeling, multilevel modeling, network analysis, and agent-based computational simulations may offer complementary strategies for testing the reciprocal mechanisms described in the present framework. Importantly, because CRTP specifies explicit relationships among measurable constructs, the theory is open to empirical confirmation, refinement, or falsification as evidence accumulates across different populations and cultural contexts.

## 7. Conclusions

The study of transgenerational trauma has evolved considerably over the past several decades, moving from early psychoanalytic interpretations toward increasingly sophisticated biological, relational, systemic, and socioecological explanations. Collectively, these perspectives have demonstrated that the consequences of trauma extend far beyond the individuals who directly experience adverse events. Yet despite substantial theoretical and empirical progress, a fundamental question has remained insufficiently addressed: why do trauma-related vulnerabilities often persist long after the original traumatic event has disappeared, the original perpetrators are no longer present, and explicit memories of the trauma have faded?

The Contextual Resonance Theory of Psychopathology was developed as an attempt to address this conceptual gap. Drawing on insights from trauma studies, attachment theory, family systems approaches, resilience research, ecological psychology, and predictive processing, the theory proposes a shift in the unit of analysis from the isolated individual to the dynamic relationship between individuals and the environments in which they develop. Rather than conceptualizing trauma transmission primarily as the movement of symptoms, memories, or biological alterations across generations, the theory suggests that trauma persists through the recursive reproduction of contexts that have themselves been shaped by traumatic adaptation.

At the center of the proposed framework lies the assumption that trauma-related responses are initially adaptive. Hypervigilance, emotional restraint, distrust, threat anticipation, and increased sensitivity to uncertainty emerge because they facilitate survival under adverse conditions. However, these adaptations do not remain confined to the individual. Over time, they become embedded within families, institutions, organizations, and communities, gradually transforming into contextual characteristics that influence how future generations learn to interpret the world. Through processes of contextual traumatic inertia and recursive contextual resonance, environments continue to communicate assumptions about safety, danger, trust, and belonging long after the original source of adversity has disappeared.

By integrating predictive processing principles, the theory further proposes that trauma survives because it continuously shapes the predictive models through which individuals anticipate reality. Contexts function as ongoing teachers of expectation. They communicate what is likely to happen, whom to trust, how much uncertainty to tolerate, and whether vulnerability is safe. In this sense, transgenerational trauma may be understood not only as the inheritance of vulnerability, but also as the inheritance of predictive environments that repeatedly reinforce particular ways of perceiving and responding to the world.

The framework also introduces corrective recontextualization as a complementary mechanism of recovery. If trauma persists through trauma-shaped environments, then healing requires more than symptom reduction or cognitive change alone. Recovery becomes possible when individuals are repeatedly exposed to contexts capable of generating alternative experiences of safety, predictability, trust, and belonging. Through sustained engagement with such environments, maladaptive predictive models may gradually be recalibrated, weakening the contextual reproduction of trauma across generations.

Ultimately, the central proposition of the Contextual Resonance Theory of Psychopathology is both simple and far-reaching: trauma may begin within individuals, but psychopathology persists through contexts. Understanding mental health therefore requires moving beyond explanations that locate suffering exclusively within the person and toward models capable of capturing the recursive interactions between minds and environments. By conceptualizing trauma as a process that becomes embedded within contexts and transmitted through contextual resonance, the present theory offers a new perspective on the persistence of suffering across generations and a new pathway for understanding how such cycles may eventually be interrupted.

If trauma can shape environments, then environments can also heal trauma. The future of trauma research may therefore depend not only on understanding what happened in the past, but also on understanding the contexts that continue to give the past its influence in the present.

## Figures and Tables

**Table 1:** Integration of theoretical traditions within the Contextual Resonance Theory of Psychopathology.

Theoretical Tradition	Core Contribution	Main Limitation	Integration within CRTP
Attachment Theory	Internal working models guide expectations about self and others.	Primarily focused on early dyadic relationships.	Internal working models are reconceptualized as context-dependent predictive schemas continuously updated through interaction with broader environments.
Family Systems Theory	Recursive interpersonal interactions maintain behavioral patterns within families.	Limited emphasis on extra-familial systems.	Recursive processes are extended to educational, institutional, organizational, and societal contexts that collectively shape psychopathology.
Ecological Systems Theory	Human development is influenced by multiple interacting environmental levels.	Primarily descriptive regarding developmental contexts.	Ecological systems become active mechanisms capable of sustaining or transforming trauma-related cognitive organization through contextual resonance.
Predictive Processing	The brain continuously generates predictions based on previous experience.	Focuses predominantly on individual cognitive processes.	Predictive mechanisms are embedded within relational and sociocultural contexts that continuously reinforce or modify expectations across generations.
Resilience Theory	Adaptation results from protective individual and contextual resources.	Often conceptualized as recovery following adversity.	Resilience is reconceptualized as the dynamic capacity of contextual systems to promote corrective reorganization through adaptive environments.
Epigenetic and Biological Models	Biological mechanisms contribute to transgenerational vulnerability.	Cannot fully explain psychological and contextual variability.	Biological predispositions are considered one interacting component within broader cognitive, relational, and contextual processes rather than independent determinants of psychopathology.

**Table 2:** Positioning the Contextual Resonance Theory of Psychopathology (CRTP) in relation to existing integrative theoretical frameworks.

Theory	Primary Unit of Analysis	Main Explanatory Mechanism	What CRTP Adds
Bandura—Reciprocal Determinism	Person–Behaviour–Environment	Reciprocal influence	Trauma-shaped contexts become autonomous adaptive systems
Sameroff Transactional Model	Child–Caregiver	Developmental transactions	Contextual continuity beyond dyadic development
Niche Construction Theory	Organism–Environment	Environmental modification	Trauma-specific contextual inheritance and recursive contextual organization
Ecological Systems Theory	Nested environments	Environmental influences	Contexts actively preserve trauma-related predictive structures
Family Systems Theory	Family	Recursive interaction	Extension to institutional and sociocultural systems

**Table 3:** Core propositions and empirically testable predictions of the Contextual Resonance Theory of Psychopathology.

CRTP Construct	Core Proposition	Testable Prediction
Contextual Traumatic Inertia	Contexts preserve trauma-related adaptations	Trauma-shaped contextual characteristics remain stable after trauma disappears
Recursive Contextual Resonance	Contexts reinforce trauma-related expectations	Threat expectations correlate with contextual threat cues
Corrective Recontextualization	Restorative contexts modify maladaptive predictions	Exposure to restorative environments predicts reduction of threat priors
Distributed Psychopathology	Vulnerability emerges across person–context systems	Contextual variables explain variance beyond individual variables

**Table 4:** Operationalization, empirical predictions, and falsification criteria for the principal hypotheses of the Contextual Resonance Theory of Psychopathology (CRTP).

Hypothesis	Variables	Comparison	Findings Supporting CRTP	Findings Challenging CRTP
Contextual traumatic inertia	family communication, institutional rigidity, perceived environmental unpredictability	Trauma-exposed communities vs. comparable non-trauma communities; longitudinal generations	Trauma-shaped contextual characteristics remain elevated after controlling for SES and current adversity	Contextual characteristics disappear rapidly after trauma or show no difference from controls
Recursive contextual resonance	attachment security, threat beliefs, interpersonal trust, contextual safety	Longitudinal repeated assessments	Reciprocal cross-lagged influences between context and cognitive expectations	Only one-way influence or absence of reciprocal effects
Distributed psychopathology	psychological symptoms plus contextual indicators	Multilevel analysis across families, schools, organizations or communities	Contextual variables explain significant variance beyond individual characteristics	Individual-level variables fully explain outcomes after contextual factors are included
Corrective Recontextualization	contextual safety, trust, emotion regulation, psychological symptoms	Intervention vs. comparison contexts	Safer environments predict progressive recalibration of threat expectations and symptom reduction	Contextual improvement produces no measurable cognitive or psychological recalibration

## Data Availability

The original contributions presented in this study are included in the article. Further inquiries can be directed to the corresponding author.
